# Understanding parental bonding in the first two years after birth: exploring family predictors using growth mixture modeling

**DOI:** 10.1186/s40359-026-04788-9

**Published:** 2026-05-27

**Authors:** Stefanie Unger, Victoria Weise, Judith T. Mack, Ariane Göbel, Andreas Staudt, Susan Garthus-Niegel

**Affiliations:** 1 Institute and Policlinic of Occupational and Social Medicine, Faculty of Medicine, University Hospital Carl Gustav Carus, TUD Dresden University of Technology, Dresden, Germany; 2 Department of Child and Adolescent Psychiatry, Faculty of Medicine, University Hospital Carl Gustav Carus, TUD Dresden University of Technology, Dresden, Germany; 3 Institute for Systems Medicine, Faculty of Medicine, Medical School Hamburg, Hamburg, Germany; 4 Department of Child and Adolescent Psychiatry and Psychotherapy, University Medical Center Hamburg-Eppendorf, Hamburg, Germany; 5 Department of Childhood and Families, Norwegian Institute of Public Health, Oslo, Norway

**Keywords:** Bonding, Depression, Parental mental health, Family, Child temperament, Birth experience, Growth mixture model, DREAM study, Relationship satisfaction

## Abstract

**Background:**

Parental bonding towards the child holds substantial importance in child development and the mental well-being of parents and children alike. Therefore, it is crucial to gain insights into the diverse trajectories that bonding difficulties may follow during the postpartum period. This study addresses a research gap by exploring the trajectories of both maternal and paternal bonding difficulties with the inclusion of various potential predictor variables of trajectory class membership in a large community-based sample.

**Methods:**

This study utilized data from the longitudinal cohort study DREAM to investigate the trajectories of maternal (*n* = 1,761) and paternal (*n* = 1,128) bonding difficulties at eight weeks, 14 months, and two years postpartum. To identify distinct trajectories of bonding difficulties, Growth Mixture Modeling was employed. Additionally, several potential predictors of trajectory class membership were examined through multinomial logistic regression, including parental mental health factors, subjective birth experience, difficult child temperament, interpersonal factors, and sociodemographic characteristics.

**Results:**

Three distinct bonding difficulty trajectory classes emerged in both maternal and paternal samples: “low-steady” (persistently low difficulties), “recovering” (initially high but decreasing difficulties), and “aggravating” (clinically significant and increasing difficulties). Parents in the “recovering” and “aggravating” classes not only encountered clinically significant bonding difficulties at some point during the study period, but also experienced fluctuating bonding difficulties over time. Multinomial logistic regression analysis also revealed various predictors of trajectory class membership. For mothers, significant predictors included anger/hostility symptoms, subjective birth experience, and difficult child temperament. For fathers, predictors included subjective birth experience, difficult child temperament, first-time parenthood, and age.

**Conclusion:**

This study challenges the notion of bonding as a stable phenomenon through the identification of non-steady trajectories, acknowledging the importance of recognizing that clinical bonding difficulties tend to manifest in non-steady courses over time. Recognizing these diverse trajectories of bonding difficulties and their predictors offers valuable insights for the development of more effective interventions and support systems to promote healthy parent-child bonding.

**Supplementary Information:**

The online version contains supplementary material available at 10.1186/s40359-026-04788-9.

## Background

Parental bonding towards their child has increasingly become a focal point of peripartum and family psychology research in recent decades, being discussed as a significant factor impacting child development and the mental health of both parents and children [1–4]. The term parental bonding, as defined by Condon [5] and Bretherton [6], pertains to the emotional connection parents form with their child and, as a concept, should be distinguished from observable behavior, such as sensitivity [7, 8], as well as the child’s attachment to their parents [9–11]. Bonding can be conceptualized as a continuous process, with parents typically forming a stable emotional connection over time [9]. However, deviations from this process – characterized by persistent difficulties in developing a positive emotional bond – can be referred to as bonding difficulties, which can vary in severity. According to Brockington et al. [12], bonding difficulties and disorders can result in hostile and aggressive parental behavior, emotional frustration or absence during interactions, as well as pathological ideation and potential rejection of the child.

Previous research has shown that parental bonding is already established during pregnancy [1, 13, 14] and tends to remain steady during the transition from pregnancy through the postpartum period [15, 16]. Regarding mothers, Faisal-Cury et al. [15] examined 346 mothers with a heightened risk of postpartum depression, discovering that bonding difficulties at 6 to 8 months predicted bonding difficulties at 12 to 15 months. For fathers, Condon et al. [13] found consistent bonding levels for 204 first-time fathers from pregnancy to one year postpartum. Furthermore, de Cock et al. [1] examined parental bonding within a Dutch community sample comprising 335 mothers and 261 fathers at the 26th week of pregnancy, as well as at six and two years postpartum. Their findings unveiled a moderate positive correlation between pre- and postpartum bonding for both parents, suggesting that initial levels of bonding during pregnancy can influence subsequent bonding dynamics. However, the presence of a moderate correlation also implies the potential for changes over time, possibly influenced by additional variables, and although bonding appears to be a relatively stable phenomenon, it is important to note that differential trajectories may exist.

The present study applies Growth Mixture Modeling (GMM) to examine heterogeneity in parental bonding trajectories, thereby extending prior research that has primarily used variable-centered or simpler person-centered methods [4, 17, 18]. GMM represents a methodologically advanced approach that aligns with contemporary developmental science [19, 20], recognizing that developmental processes are inherently heterogeneous and that averaging across individuals can obscure meaningful subgroups with distinct bonding patterns [21, 22]. By doing so, GMM enables a more nuanced understanding of bonding development over time.

To date, no study has applied GMM to investigate parental bonding. De Cock et al. [14] conducted a longitudinal latent class analysis (LCA) using bonding scores from pregnancy, six months, and two years postpartum as indicators. By capturing interindividual differences in bonding patterns, this represented an important methodological step beyond traditional mean-based longitudinal approaches, which examine only average trends across the sample. However, LCA remains limited to grouping individuals based on similar response profiles across time points. GMM, in contrast, models both the form and rate of change – allowing for linear and nonlinear trajectories – and thus provides a more comprehensive account of developmental heterogeneity in bonding. De Cock et al. [14] therefore identified latent bonding patterns based primarily on mean-level differences (intercepts) rather than changes in slope across the three assessment points. Their results indicated relatively stable, parallel patterns of bonding from pregnancy through toddlerhood. However, the study was limited by a relatively small sample size (*n* = 370 mothers, *n* = 292 fathers) and only two postpartum measurement points within the first two years. The present study addresses these limitations by applying GMM to a large community sample assessed at three postpartum time points, thereby enhancing parameter estimation and enabling the creation of more versatile and intricate models [23–25].

Two prior variable-centered studies have examined changes in parental bonding over time, although both focused exclusively on mothers. Roth et al. [17] used linear multilevel modeling across the first six months postpartum and found modest mean-level increases in bonding (β = 0.13 per month). Garon-Bissonnette et al. [18] also applied multilevel modeling to changes in bonding across the first seven weeks postpartum. Both studies highlighted links between parental distress (depression, anxiety) and bonding, underscoring the importance of early identification and intervention on modifiable factors such as depression management, breastfeeding support, and mindfulness. However, neither study examined qualitatively distinct developmental patterns, underscoring the value of a person-centered approach such as GMM to identify longitudinal differences among otherwise unobserved subgroups.

From a clinical perspective, identifying differential bonding trajectories offers important preventive and translational insights. While GMM findings are primarily exploratory and not directly applicable for intervention, a trajectory-based understanding of bonding development can inform more targeted, group-specific prevention strategies rather than one-size-fits-all approaches [20]. Because bonding trajectories during the first two years are closely linked to children’s long-term mental health and developmental outcomes, early identification of at-risk trajectory classes has considerable preventive relevance [2, 26].

While bonding has traditionally been studied in mothers, less attention has been given to paternal bonding. However, findings on maternal bonding may not be entirely applicable to fathers, given differences in their roles in aspects such as breastfeeding and childcare, as well as unique parent-specific behaviors. This has been exemplified by activities like stimulatory play being more frequent in father-child interactions, while affectionate behaviors were observed to be more prevalent in mother-child interactions [27, 28]. These parent-specific behaviors have also been associated with unique differences in brain activation patterns observed during childcare [29]. Given that maternal and paternal bonding may differ in their underlying mechanisms and the socialization experiences that contribute to their development, particularly regarding distinct roles and behaviors during childcare, it is reasonable to expect that the correlates or predictors of bonding may differ between mothers and fathers. Therefore, it is crucial to investigate these potential differences to gain a comprehensive understanding of the factors that influence parental bonding. To achieve this, we developed separate models to uncover unique trajectories in maternal and paternal bonding. Furthermore, we incorporated an extensive set of variables as predictors for membership in the trajectory classes, including many that have received minimal or no prior attention in the context of paternal bonding research. This approach empowers us to explore the distinctions between these trajectory classes with respect to the predictor variables. Previous research has studied factors that are related to or have an influence on parental bonding [11, 28]. Of those, parental mental health factors, the subjective birth experience, difficult child temperament, as well as family and sociodemographic characteristics are particularly noteworthy.

### Parental mental health factors

The peripartum period is commonly recognized as a potentially challenging time during which parents may face a range of mental health difficulties or struggles [30–33], with up to 15% of mothers and 9% of fathers experiencing depressive symptoms [34–36]. In a meta-analysis by O’Dea et al. [37], depression exhibited the strongest correlation with maternal bonding among the various examined domains of maternal distress, which included anxiety symptoms, stress symptoms, and postpartum blues. This corroborates findings from other studies indicating that peripartum depressive symptoms in parents may contribute to bonding difficulties [38–42].

Anxiety symptoms are often studied in relation to parental bonding. Research has shown that approximately 2.6 to 39% of mothers and 4.1 to 16% of fathers experience anxiety symptoms during pregnancy [43, 44], indicating a high prevalence among expectant parents. A study suggests that mothers report more severe anxiety symptoms than fathers during both the pre- and postpartum periods [45], which may have important implications for the development of bonding to their child. Indeed, high levels of prepartum maternal anxiety symptoms have been linked to more bonding difficulties pre- and postpartum [14, 46–51], while limited research has shown a similar effect for fathers [14].

Similarly, numerous studies have linked the onset or worsening of obsessive-compulsive symptoms (OCD symptoms) during pregnancy in women to poorer postpartum adaptation and maternal bonding and attachment issues [52–56]. Although many parents experience disturbing intrusive thoughts about harming their newborn during the peripartum period, only a few develop clinically significant OCD symptoms [57, 58]. While the prevalence of OCD symptoms in mothers is reported lower during pregnancy (0.2% to 3.5%) compared to the postpartum period (2.7% to 9%;) [58, 59], for fathers, Coelho et al. [60] found a prevalence of 3.4% during pregnancy and 1.8% postpartum. Peripartum research has predominantly focused on maternal OCD symptoms, highlighting the burden of compulsions and intrusive thoughts, which can significantly impact maternal well-being and daily functioning [57, 61–63]. Limited exploration exists regarding fathers’ experiences with OCD symptoms during the peripartum period, although Blum et al. [64] found no significant link between parental OCD symptoms at eight weeks postpartum and parental bonding after controlling for confounders. Despite some evidence suggesting prepartum OCD symptoms adversely affect postpartum bonding for both parents, definitive conclusions are hindered by the scarcity of studies, particularly from the fathers’ perspective.

Although the relationship between prepartum somatization symptoms and parental bonding has not been fully established, some studies suggest a potential link between these variables. Pregnant women often report higher levels of somatization symptoms than their partners [65] and non-pregnant women [55, 66]. Those with particularly high levels of somatization are at increased risk of maternal distress [67] and postpartum depression, which can, in turn, negatively impact bonding [68]. As such, prepartum somatization symptoms may exert a negative impact on postpartum bonding among parents, although there is limited research on fathers’ somatization symptoms during the peripartum period.

Several studies have shown that the peripartum period can be a time of heightened emotional distress for parents, which may include feelings of anger/hostility [69, 70]. While numerous studies have examined maternal anger/hostility symptoms during pregnancy, research on the paternal perspective remains scarce. A study by Göbel et al. [71] found that maternal anger/hostility symptoms had a detrimental effect on bonding, whereas no such effect was observed in the fathers. Moreover, maternal anger/hostility symptoms have shown to be associated with increased levels of frustration with the child [72] and perceived stress during pregnancy [73], which has been linked to postpartum bonding difficulties [74]. Mäntymaa et al. [75] discovered a link between maternal anger/hostility symptoms in early mother–infant interaction and subsequent behavioral and emotional problems in the child. In the assessment of anger among fathers, Francis et al. [76] found that trait anger in fathers was positively associated with parenting stress, partially mediated by patience and tolerance – key aspects of paternal bonding. Similarly, Macdonald et al. [77] reported that fathers with higher anger levels had lower perceived social support and weaker co-parenting relationships, marked by lower agreement on parenting goals, unequal childcare division, and increased conflict. Remarkably, severe depression and physical anger were strongly associated with diminished paternal bonding towards their toddler. Research indicates that maternal and paternal anger/hostility symptoms may have differential effects on bonding, yet there is a scarcity of studies concerning male anger/hostility within the parenting context.

### Subjective birth experience

Besides mental health factors, researchers have studied the subjective birth experience as a predictor for bonding, mostly for mothers. Studies have linked a better maternal birth experience to less maternal bonding difficulties [78, 79]. Conversely, a negative birth experience was linked to poorer parent-child bonding two years postpartum in both parents [80]. Negative birth experiences have also been associated with mothers perceiving the newborn’s behavior as distant and intrusive [81] along with lower maternal self-esteem and self-efficacy [82]. Moreover, a negative birth experience can also increase the risk of developing childbirth-related post-traumatic stress symptoms [83, 84], which in turn can cause bonding difficulties [80, 85–88], although findings are mixed [45, 88–90]. Recent findings by Seefeld et al. [80] have highlighted the influence of the subjective birth experience on parental bonding in both mothers and fathers. Despite the general alignment between mothers’ and fathers’ perceptions of the birth experience [91], research on the fathers’ perspective is still sparse, compared to the existing body of literature on the mothers. In this study, we seek to contribute further insights into how fathers’ subjective birth experiences influence paternal bonding.

### Difficult child temperament

Child temperament plays a critical role in the development of parental bonding, with difficult child temperament being linked to parental bonding difficulties [14, 45, 92, 93]. Although many studies have investigated the relationship between concurrent measures of difficult child temperament and parental bonding [45, 92] or the prospective impact of difficult child temperament on maternal bonding [93], some studies further suggest that impaired parental bonding could have negative effects on how parents perceive their child’s temperament. For instance, previous research has demonstrated that postpartum parental bonding difficulties are linked to negative affectivity in children at the age of two years [14, 94]. Additionally, maternal bonding measured 14 months postpartum was found to be a significant predictor of a child’s behavioral problems at 5.5 years of age, even after accounting for maternal psychopathology [95]. These findings suggest a persistent and bidirectional nature of the relationship between parental bonding and difficult child temperament, with a more difficult child temperament being associated with a greater likelihood of bonding difficulties in both parents.

### Interpersonal factors

Transitioning from pregnancy to becoming a family with a new child presents many challenges, including more parental conflicts [96–99]. Low parental relationship satisfaction is a critical factor in the adjustment process and can threaten parental mental health, whereas high satisfaction may serve as a protective factor [70, 100, 101]. Research has further shown that relationship satisfaction during pregnancy impacts postpartum parental bonding, especially in fathers [45, 102, 103]. Finally, greater partner support in both parents has been linked to reduced pregnancy stress and fewer bonding difficulties [104, 105].

Despite its impact on bonding not being clearly established, we will consider the potential role of first-time parenthood on bonding. For the context of this study, ‘first-time parenthood’ refers to mothers who have not previously given birth and fathers who have not previously experienced fatherhood with at least one biological child before the pregnancy with the index child. The arrival of a new child often leads to higher stress levels for parents [96, 97], resulting in more parental conflicts [98] and difficulties in dealing with their children’s behavior [106, 107]. Siblings may also find this transitional phase challenging, with their behavior often being perceived as more problematic [107]. Consequently, they may require additional support [108–110], potentially increasing the psychological burden on parents. In this context, it is also conceivable that parents may find it more difficult to emotionally focus on the second (or subsequent) child to the same extent as they did with their firstborn. As a result, it is reasonable to assume that parents with preexisting children may face greater bonding difficulties than first-time parents.

### Sociodemographic characteristics

Our study aims to comprehensively examine factors that may influence bonding by analyzing sociodemographic variables identified as potential predictors in previous research. For the impact of parents’ age on bonding research indicates mixed findings. While de Cock et al. and Hall et al. [14, 16] suggest that older parents may encounter greater bonding challenges, their findings diverge notably in terms of gender differences. Specifically, de Cock et al. [14] observed a significant impact of age on maternal bonding but not paternal bonding, whereas Hall et al. [16] found the opposite, with age showing a significant impact on paternal bonding but not maternal bonding. Moreover, Bicking Kinsey et al. [111] highlighted a correlation between higher maternal age and bonding difficulties. Overall, previous research underscores the potential link between higher parental age and postpartum bonding difficulties. Additionally, parental education has been identified as a potential factor influencing bonding. Several studies have indicated a link between higher educational levels and bonding difficulties among mothers [39, 105, 111] as well as both parents [14]. Bohne et al. and Hall et al. [16, 112] identified the same positive association between higher educational levels and bonding difficulties in fathers, but found no such effect in mothers.

### Study aims and hypotheses

In this study, our primary objective was to assess the longitudinal trajectories of maternal and paternal bonding separately over three distinctive timepoints: eight weeks, 14 months, and two years postpartum. Drawing from previous research, we anticipate identifying distinct groups of mothers and fathers with varying trajectories of bonding, which will be influenced by the extent of bonding difficulties the parents face. We also hypothesize that certain predictor variables will have a differential impact on which trajectory class the mothers and fathers will be assigned to, respectively. The predictor variables comprise depressive, anxiety, OCD, somatization, and anger/hostility symptoms, along with subjective birth experience, difficult child temperament, relationship satisfaction, first-time parenthood, parental age, and education.

## Methods

The data for this study were obtained from the Dresden Study on Parenting, Work, and Mental Health (DREAM), an ongoing prospective cohort study that recruited expectant mothers and their partners mainly from birth clinics and midwife practices in the area of Dresden, Germany, between June 2017 and the end of 2020 (see study protocol [113] for details on recruitment and data collection). The DREAM study currently includes one prepartum (T1) and six postpartum timepoints (T2 to T7) until 7,5 years after childbirth, at which parents filled out questionnaires. Recruitment was carried out during late pregnancy through multiple community and healthcare channels to ensure a diverse sample, including obstetric clinics, midwife practices, freestanding birthing centers, early care services, gynecological outpatient settings, child-related stores, health insurance magazines, and online or word-of-mouth announcements. The present study analyzed data collected at the first four time points: during pregnancy (T1), as well as at eight weeks (T2), 14 months (T3), and two years (T4) postpartum. Specifically, T1 (late pregnancy) served as the baseline assessment; T2 (8 weeks postpartum) was scheduled after the puerperium to capture early postnatal adjustment once acute physical recovery was typically complete; T3 (14 months postpartum) corresponded to the end of the standard German parental allowance (*Elterngeld*), assuming both parents took the minimum two months each of the 14-month benefit period; and T4 (24 months postpartum) coincided with the end of the extended parental allowance (*ElterngeldPlus*) for parents who worked part-time while extending their benefits [113].

**Fig. 1:**
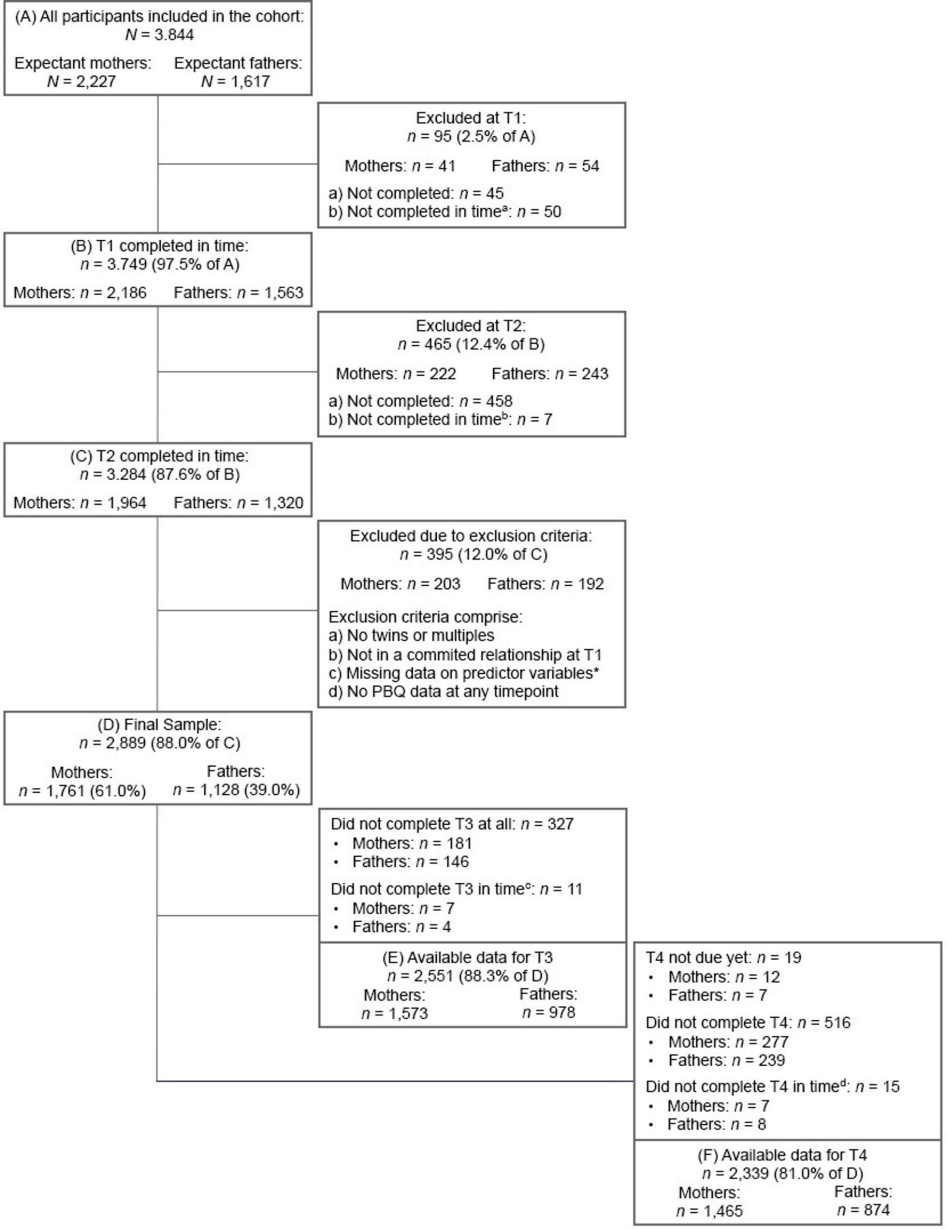
Flowchart of retention rate and exclusion criteria resulting in final sample. *Missing data pertaining sum score after imputation via mean replacement if incomplete questionnaire items were ≤ 20%. Note. T1 = during pregnancy; T2 = around 8 weeks after the anticipated birth; T3 = around 14 months after the actual birth date; T4 = around 2 years after the actual birth date. Data for the current study were extracted on March 10, 2023 (version 10 of the quality-assured data files). Prospective data collection was complete for T1, T2, and T3 and ongoing for T4. ^a^Not within pregnancy. ^b^Not within 24 weeks after the planned birth date. ^c^Not within 12 and 16 months after the actual birth date. ^d^Not within 22 and 26 months after the actual birth date

The initial sample for our study consisted of *N* = 2,227 mothers and *N* = 1,617 fathers who provided their consent to participate. To ensure a focused examination specifically on mothers and fathers, we deliberately excluded female partners of mothers (*n* = 16) from our study. This decision was made to maintain the clarity and distinctiveness of our analysis, while also acknowledging that the small group size may have limited statistical power and the ability to draw meaningful conclusions. Out of the parents who consented to participate, a total of *n* = 560 parents were excluded from the analysis because they did not complete the T1 or T2 questionnaires within our designated time criteria (designated timeframes: during pregnancy for T1, up to 24 weeks postpartum for T2, 12 to 16 months postpartum for T3, and 22 to 26 months postpartum for T4). Additionally, *n* = 83 parents with multiple pregnancies were excluded based on prior research indicating that parents of multiples experience higher levels of depression, anxiety, and parental stress during the peripartum period compared to those with singleton birth [114]. Further details and rationale for this decision are provided in Supplementary Material 3. Moreover, we excluded *n* = 23 parents who were not in a committed relationship during T1. This exclusion was necessary as these individuals would not have been able to contribute data regarding the predictor variable of relationship satisfaction. Furthermore, *n* = 289 parents were excluded due to missing data on predictor variables at T1 and T2, despite their participation at these time points. Their exclusion was necessary, as our study’s predictors were assessed at these specific time points and, in standard Mplus implementations, these cases are subject to listwise deletion because the regression step functions as a weighted multinomial logistic regression and does not perform FIML estimation for missing predictor data. Although multiple imputation of covariates prior to analysis is technically feasible, we refrained from this approach to maintain conceptual clarity and measurement validity, as most predictors represent self-reported psychological constructs. This complete-case strategy ensures that all reported associations are based on observed data and supports transparency and reproducibility. Notably, all remaining participants provided data on parental bonding at least once between T2 and T4, ensuring the completeness of bonding-related analyses. Yet, it is worth noting that, at T3 and T4, the bonding data of *n* = 11 and *n* = 15 cases respectively were not considered for analysis, as these participants did not meet our designated time criteria at those specific time points. However, data from these participants at other time points were retained in the sample. Considering these exclusion criteria, our final sample sizes consisted of *n* = 1,761 mothers and *n* = 1,128 fathers. Figure 1 provides a comprehensive breakdown of the participant flow, exclusion criteria, and dropout rates.

### Measures

The following measures were assessed to evaluate both outcome and predictor variables.

#### Bonding difficulties (Outcome)

To assess parental bonding, we administered the Postpartum Bonding Questionnaire (PBQ) [12, 115] to all parents at timepoints T2, T3, and T4. Although originally developed as a screening tool to capture maternal bonding [116], the PBQ has also been used in several studies to assess bonding from the father’s perspective [28, 117, 118]. The PBQ comprises 25 items, each scored on a six-point Likert scale, where parents indicate how often statements about their child were true for them during the worst time with their child (0 = never; 5 = always). For this study, the sum score (range: 0 to 125) was used with a higher score indicating more bonding difficulties. This approach was chosen because prior research has shown that PBQ subscale structures vary across samples, time points, and language versions [119, 120], whereas the total score demonstrates greater longitudinal stability and high internal consistency (Cronbach’s α = 0.85–0.90). Using the total score also allows for comparability across time points (T2–T4) and provides a clinically interpretable measure of bonding difficulties. A total score of ≥ 26 is considered clinically significant for bonding difficulties, according to Brockington et al. [10]. In our sample of mothers, we obtained excellent internal consistencies (Cronbach’s α = 0.90) at all three timepoints. For fathers, the internal consistencies acquired were α = 0.85 (T2), α = 0.87 (T3), and α = 0.88 (T4).

#### Depressive symptoms

Depressive symptoms were assessed at T1 using the German version of the Edinburgh Postnatal Depression Scale (EPDS) [121]. The EPDS is a screening tool initially developed to assess postpartum depressive symptoms in mothers. The 10 items contain statements about feelings and thoughts experienced by the participant in the last seven days, rated on a four-point scale (ranging from 0 to 3). Items where higher scores indicate lower levels of depressive symptoms were reversed such that higher scores reflect higher levels. The total sum score of all items (0–30) was utilized for analysis. In our samples, the internal consistency was α = 0.83 for mothers and α = 0.81 for fathers.

#### Anxiety, OCD, somatization, and anger/hostility symptoms

To assess anxiety, OCD, somatization, and anger/hostility symptoms, the Symptom Checklist-90-Revised (SCL-90-R) [122] was utilized at T1. The SCL-90-R is a 90-item self-report questionnaire designed to assess psychological and physiological symptoms and discomfort across nine symptom dimensions and has been previously utilized during the peripartum period and in other research [123–125]. However, for the purpose of this study, only the aforementioned dimensions were utilized. Participants rated the severity of the described symptoms experienced over the past seven days on a five-point Likert scale, ranging from “not at all” (0) to “very much” [4]. The sum scores of the four respective subscales were calculated for data analysis. The internal consistencies for the anxiety subscale were α = 0.74 for mothers and α = 0.68 for fathers, for the OCD subscale α = 0.79 and α = 0.81, for the somatization subscale α = 0.76 and α = 0.78, and for the anger/hostility subscale α = 0.70 and α = 0.73.

#### Subjective birth experience

To assess the subjective birth experience of participants, the German version of the Salmon’s Item List (SIL) [126], a validated 20-item questionnaire consisting of four dimensions: fulfillment, physical discomfort, emotional distress, and negative emotional experience, was administered at T2. Although the SIL was initially designed for mothers to evaluate their childbirth perceptions and experiences, it has also proved suitable as an instrument for assessing fathers’ birth experiences [127, 128]. Parents were asked to rate each item on a scale of 0–6, based on their feelings during childbirth. Prior to analysis, negatively worded items were recoded so that for all items, a higher score indicated a more positive subjective birth experience. In this study, the total sum score, ranging from 0 to 120, was used for analysis. Internal consistencies for the SIL were excellent, with α = 0.91 for mothers and α = 0.86 for fathers.

#### Difficult child temperament

Difficult child temperament was assessed at T2 using the “fussy-difficult” subscale of the Infant Characteristic Questionnaire (ICQ), a brief screening tool developed by Bates et al. [129]. It comprises 9 items containing statements about the child’s typical behavior, which can be rated on a seven-point Likert scale by parents or individuals close to the child. The scale ranges from 1 (easy temperament) to 7 (difficult temperament), with 4 representing a mid-range temperament. The “fussy-difficult” scale exhibits the highest internal consistency among all the subscales in the ICQ (0.79 for women, standardized alpha according to Bates et al.’s original study). The overall value of the ICQ “fussy-difficult” scale was obtained as a sum score, with the lowest attainable score being 9 and the highest being 63. In our study, we obtained very satisfactory internal consistencies of α = 0.87 for mothers and α = 0.86 for fathers.

#### Relationship satisfaction

To measure relationship satisfaction, we utilized the short form of the Partnership Questionnaire (PFB-K) created by Kliem et al. [130] at T1. The PFB-K comprises three items from each of the three subscales of the Partnership Questionnaire (FPD) [131]. The nine items contain statements about the partner’s or the couple’s behavior in the relationship. Parents can rate the frequency of the different behaviors on a four-point Likert scale ranging from 0 (never/very rarely) to 3 (very often). For this study, all negatively phrased items have been recoded so that a higher value indicates higher relationship satisfaction for all items. The overall score of the PFB-K will be utilized in the present study, with a minimum possible score of 0 and a maximum possible score of 27. In this study, we found internal consistencies of 0.81 for mothers and 0.80 for fathers.

#### First-time parenthood, parental age, and education

To determine whether the mothers and fathers in this study were first-time parents, the item “How many children do you have?” was developed and answered numerically, excluding the unborn offspring. For fathers, being a first-time parent specifically referred to biological children. The item was adapted from the Norwegian Akershus Birth Cohort (AHUS) study, where it has deemed effective for assessing parental parity in large population samples [132]. We employed self-developed items to assess parental age and education. Education level was assessed using the item “What is your highest level of education?” which included various options representing common types of school graduation in Germany. For the analysis, education level was dummy-coded to distinguish individuals with more than 10 years of school education from those with 10 years or less.

### Data analysis

Data analysis was conducted in SPSS version 27.0 for demographic analysis, internal consistency calculations, multicollinearity checking, detection of potential multivariate outliers based on the Mahalanobis distance method, as well as attrition analyses. Mean replacement was applied at the individual level, such that when no more than 20% of items within a questionnaire were missing, missing values were replaced by the participant’s mean score on the completed items of that questionnaire. Given this threshold (≤ 20%), variance attenuation at the composite score level is expected to be minimal, consistent with simulation findings suggesting unbiased estimates under low rates of missing item data [133].

Distinct bonding trajectories in both the maternal and paternal samples were identified using Mplus version 7.31 [134] through the development of two GMMs. This modeling approach enables the detection of longitudinal differences in measured variables, specifically parental bonding between T2 and T4, among unobserved subgroups within the sample [135]. In this analysis, repeated measures were treated as manifest indicators of latent growth over time. The continuous growth factors captured the longitudinal trajectory, while the latent subgroups were derived through a categorical class variable. The primary objective of this method was to categorize a heterogeneous sample into homogeneous subgroups based on their trajectories of bonding difficulties over time. Determining the optimal number of classes for the best-fit model involved considering both mathematical indicators and pragmatic strategies. Mathematical indicators such as the Bayesian Information Criterion (BIC) [136], Sample-Size adjusted BIC, Akaike Information Criterion (AIC) [137], Bootstrap Likelihood Ratio Test (BLRT), and Lo, Mendell, and Rubin Likelihood Ratio Test (LMR-LRT) [138] were utilized to guide this decision-making process.

To identify the optimal model parameters, we systematically tested models with varying numbers of latent classes to determine the best overall fit. After determining the optimal number of classes, predictors of class membership were incorporated using the regression-based three-step (R3STEP) procedure implemented in Mplus [139]. The R3STEP procedure offers key advantages over the traditional one-step approach, as it prevents covariates from influencing the latent class formation and preserves the measurement integrity of the underlying mixture model.

The 3-step method involves three steps: First, a GMM is estimated without predictors to identify the latent classes. Second, a nominal variable N indicating the most likely class membership for each participant is developed based on the estimation from the first step. Finally, multinomial logistic regression is performed, treating N as the dependent variable and incorporating the predictor variables as independent variables, while considering the probabilistic class assignment from the second step. This approach avoids biases introduced by including or excluding covariates and ensures accurate estimation of latent classes. The final model includes the observed bonding data (PBQ) at T2–T4, the latent class variable, and the growth parameters intercept and slope. To account for individual missing data points in the PBQ assessment at timepoints T2–T4, the Full-Information Maximum-Likelihood (FIML) procedure was utilized. Rather than imputing specific data points, FIML estimates statistical parameters based on the variance-covariance matrix of the sample, optimizing the fit to the available dataset [140]. This approach is comparable to Multiple Imputation (MI) in effectively addressing missing data points [141].

#### Gender-wise class comparison

Following the initial GMM analysis, we conducted an additional gender-wise class comparison to explore potential differences between mothers and fathers. This analysis was not part of the original study design but was added post hoc after observing similarities in their trajectory classes, allowing for a more nuanced interpretation of the findings. For that purpose, two nested multi-group models were compared using the Satorra-Bentler scaled chi square test [142]. The first model included equality constraints and assumed that bonding trajectories in the latent classes were equal in mothers as well as in fathers. In the second model, the trajectories were allowed to be freely estimated to examine whether allowing for gender-specific differences improved model fit, thereby assessing the necessity of differentiating bonding trajectories by parent gender. Next, we tested differences in the latent growth factors between mothers and fathers within each latent class separately to determine whether the amount (intercept) or development (slope) of bonding difficulties varied by gender within each class, providing further insight into potential gender-specific patterns.

#### Multicollinearity analysis

To assess multicollinearity as a prerequisite for the main analyses, we calculated the correlations between all the potential predictors for the maternal and paternal sample, respectively (see Supplementary Tables 1 and 2, Additional file 1). Correlation coefficients exceeding |r| = 0.7 are commonly considered indicative of multicollinearity [143]. Although no correlations reached this threshold in our data, several predictors showed moderate intercorrelations above |r| = 0.5, which prompted sensitivity analyses to examine potential overlap in explained variance due to conceptual redundancy, masked associations, or suppression effects (see Supplementary Tables 3 and 4, Additional file 1). These analyses led us to exclude the following variables from subsequent models: in the maternal model, OCD symptoms were omitted, and in the paternal model, both OCD symptoms and education were excluded.

#### Outlier detection

To identify multivariate outliers, Mahalanobis distances (MD) were calculated separately for mothers and fathers based on the full predictor set. Although MD is a widely used method for detecting multivariate outliers, it is known to have limitations such as lack of robustness and resistance to the influence of outliers [144, 145]. Nonetheless, it remains a widely used and informative method for identifying cases with multivariate predictor profiles that substantially deviate from the sample distribution. To mitigate these limitations, we combined a chi-square-based threshold with visual inspection of the MD distribution to ensure a conservative and justified exclusion of cases. Based on this procedure, five mothers and ten fathers were excluded. A detailed description of the outlier detection and exclusion process, along with the sensitivity analyses results, is provided in Additional file 2.

## Results

### Sample characteristics

Descriptive characteristics of the samples are presented in Table 1. Parents completed the first questionnaire T1 around the end of the second trimester (M = 29.53 gestational weeks, SD = 6.52 for mothers; M = 29.86 gestational weeks, SD = 6.52 for fathers). At that time, mothers were on average 30.20 years old (SD = 3.94), whereas fathers were slightly older with a mean age of approximately 32 years (M = 32.41, SD = 4.87). The majority of participants were well-educated, with 78.5% of mothers and 72.6% of fathers having received more than 10 years of education. Furthermore, 80.4% of mothers and 80.5% of fathers expected their first child, and the great majority held German citizenship (97.0% of mothers and 98.6% of fathers).

**Table 1:** Sample characteristics

	Mothers		Fathers	
	M (SD)	Range	M (SD)	Range
Parental age (in years; T1)	30.20 (3.94)	18–43	32.41 (4.87)	20–56
Gestational week at T1	29.53 (6.52)	8–41	29.86 (6.52)	8–41
Bonding scores (PBQ score; T2 – T4)				
Parent-child bonding at T2	12.83 (9.84)	0–93	12.57 (8.41)	0–54
Parent-child bonding at T3	13.69 (9.95)	0–102	13.36 (8.38)	0–68
Parent-child bonding at T4	14.38 (9.75)	0–80	13.75 (8.69)	0–68
Predictor variables				
Depressive symptoms (EPDS; T1)	5.52 (4.00)	0–27	3.76 (3.58)	0–26
Anxiety symptoms (SCL-90-R; T1)	2.51 (3.09)	0–23	1.76 (2.69)	0–27
Obsessive-compulsive symptoms (SCL-90-R; T1)	4.41 (4.01)	0–32	3.23 (3.81)	0–29
Somatization symptoms (SCL-90-R; T1)	5.59 (4.60)	0–32	2.37 (2.83)	0–22
Anger/hostility symptoms (SCL-90-R; T1)	2.03 (2.13)	0–14	1.45 (2.14)	0–20
Subjective birth experience (SIL; T2)	78.49 (20.76)	6–120	92.78 (15.12)	23.33–120
Difficult child temperament (ICQ; T2)	28.43 (8.01)	9–55	28.86 (7.73)	10–54
Relationship satisfaction (PFB-K, T1)	21.70 (4.02)	2–27	20.68 (4.15)	5–27

	***n*** ^**a**^	**%** ^**b**^	***n*** ^**a**^	**%** ^**b**^
First-time parent				
No prior children	1,412	80.4	908	80.5
At least 1 older child	288	16.4	167	14.8
More than 1 older child	56	3.2	53	4.7
Education				
Up to 10 years	378	21.5	309	27.4
More than 10 years	1,378	78.5	819	72.6
Marital status				
Married	784	44.7	531	47.1
Other	968	55.3	597	52.9
Citizenship				
German	1,704	97.0	1,112	98.6
Other	52	3.0	16	1.4
Country of birth				
Germany	1,683	96.0	1,096	97.5
Other	70	4.0	28	2.5
Clinical bonding difficulties (T2 – T4)				
PBQ ≥ 26 (T2)	158	9.0	79	7.0
PBQ ≥ 26 (T3)	156	10.0	76	8.0
PBQ ≥ 26 (T4)	146	10.0	69	8.0

*PBQ* Postpartum Bonding Questionnaire, *EPDS* Edinburgh Postnatal Depression Scale, *SCL*-*90*-*R* Symptom Checklist-90-Revised, *SIL* Salmon’s Item List, *ICQ* Infant Characteristic Questionnaire, *PFB*-*K* Short version of the Partnership Questionnaire, *T1* During pregnancy, *T2* Eight weeks postpartum, *T3* 14 months postpartum, *T4* Two years postpartum

^a^Slight variances in n due to missing data among certain participants

^b^Valid percent

Regarding bonding difficulties, the mean scores of the Postpartum Bonding Questionnaire (PBQ) varied from 12.83 (SD = 9.84) at T2 to 14.38 (SD = 9.75) at T4 for mothers, and from 12.57 (SD = 8.41) at T2 to 13.75 (SD = 8.69) at T4 for fathers. For context, at T2, 9.0% of mothers and 7.0% of fathers reported clinical levels of bonding difficulties. Over time, these percentages slightly increased, with 10.0% of mothers and 8.0% of fathers reporting clinical levels of bonding difficulties at T2 and T3. When comparing our sample’s bonding scores to those published by Reck et al. [115] for non-depressed and depressed mothers (M = 9.07 and M = 12.13, respectively), our sample reported somewhat higher levels of bonding difficulties. However, our results align closely with the mean score of M = 12.3 reported by Brockington et al. [12] for non-depressed mothers without bonding disorders. Moreover, our sample’s mean bonding scores generally indicate relatively low levels of bonding difficulties, as established by Brockington et al. [10].

### Attrition analyses

We conducted attrition analyses to compare the two final samples with participants excluded due to missing predictor variable data at T1 and T2, who otherwise met our criteria for inclusion. There were no significant differences between the two groups except from three variables (see Supplementary Tables 1 and 2, Additional file 4). Amongst mothers, the final sample showed lower levels of anger/hostility symptoms compared to the excluded mothers, *t* = -2.120, mean difference = -0.568, SE = 0.268, 95% CI [-1.098, -0.038], *p* = .036 for unequal variances. For fathers, the final sample was approximately one year younger than the excluded fathers, *t* = -2.567, mean difference = -1.084 years, SE = 0.422, 95% CI [-1.912, -0.256], *p* = .010 for equal variances. Additionally, the final sample reported lower symptoms of depression than the excluded fathers, *t* = -2.014, mean difference = -0.593, SE = 0.294, 95% CI [-1.170, -0.015], *p* = .044 for equal variances.

### Growth mixture model: model setup

To identify optimal model parameters for our analysis, we assessed the model’s fit by varying class sizes, with the mathematical model fit parameters for each class number presented in Table 2 (mothers) and Table 3 (fathers). Class counts, estimated proportions, average posterior probabilities, and visual representations of the 1- to 5-class GMM solutions for both the maternal and paternal samples are provided in Additional File 5.

**Table 2:** Model fit for k = 1–5 classes in the maternal sample

								Class sizes		A-posteriori probabilities	
k	AIC	BIC	saBIC	saBIC Δ	Entropy	LMR-LRT	BLRT	Min	Max	Min	Max
						*p*	*p*	*N*	*N*	prob	prob
1	33404.343	33448.109	33422.694	NA	NA	NA	NA	1,756	1,756	1	1
2	32918.465	32978.644	32943.698	478.996	0.966	0.000	0.000	75	1,681	0.925	0.994
3	32566.839	32643.431	32598.954	344.744	0.964	0.001	0.000	55	1,640	0.901	0.991
4	32455.937	32548.940	32494.933	104.021	0.916	0.338	0.000	25	1,523	0.821	0.970
5	32332.883	32442.298	32378.760	116.173	0.920	0.289	0.000	8	1,493	0.808	0.974

*AIC* Akaike Information Criterion, *BIC* Bayesian Information Criterion, *saBIC* Sample-Size Adjusted BIC, *saBIC Δ* Difference in sample-size adjusted BIC between adjacent class solutions (k – 1 → k), *LMR*-*LRT* Lo, Mendell, and Rubin Likelihood Ratio Test, *BLRT* Bootstrap Likelihood Ratio Test

**Table 3:** Model fit for k = 1–5 classes in the paternal sample

								Class sizes		A-posteriori probabilities	
k	AIC	BIC	saBIC	saBIC Δ	Entropy	LMR-LRT	BLRT	Min	Max	Min	Max
						*p*	*p*	*N*	*N*	prob	prob
1	19489.408	19529.563	19504.153	NA	NA	NA	NA	1,118	1,118	1	1
2	19280.973	19336.186	19301.247	202.906	0.947	0.177	0.000	41	1,077	0.879	0.991
3	19106.925	19177.195	19132.728	168.519	0.932	0.004	0.000	32	1,030	0.858	0.980
4	19051.964	19137.292	19083.296	49.432	0.910	0.034	0.000	14	999	0.795	0.970
5	19022.604	19122.990	19059.465	23.831	0.895	0.403	0.000	6	973	0.771	0.955

*AIC* Akaike Information Criterion, *BIC* Bayesian Information Criterion, *saBIC* Sample-Size Adjusted BIC, *saBIC Δ* Difference in sample-size adjusted BIC between adjacent class solutions (k – 1 → k), *LMR*-*LRT* Lo, Mendell, and Rubin Likelihood Ratio Test, *BLRT* Bootstrap Likelihood Ratio Test

Model selection followed a sequential evaluation of fit indices, consistent with recommended practice [21, 146]. Information-based criteria (AIC, BIC, saBIC) were examined first, with smaller values indicating a better balance between model fit and parsimony; the point at which the decrease in values levelled off across solutions was used to inform the optimal class number. Subsequently, likelihood ratio tests (LMR-LRT and BLRT) were consulted, comparing each k-class solution to the k − 1 class solution, with significant p-values (< 0.05) indicating a meaningful improvement in fit. Finally, entropy and average posterior probabilities were examined to assess the precision of class assignments, alongside the substantive interpretability and distinctiveness of the identified trajectory classes.

Regarding mothers, we opted for the 3-class solution due to its strong and convergent performance across all criteria. Information-based criteria showed a substantial drop in AIC, BIC, and saBIC up until k = 3, with values levelling off thereafter, indicating the 3-class solution as the optimal balance between model fit and parsimony. The model further yielded significant LMR-LRT and BLRT p-values (*p* = .001; *p* < .001), signifying a strong fit to the data, high entropy (0.964), and very satisfactory posterior probabilities (0.901–0.991), reflecting high confidence in class assignments. The rejection of 4- and 5-class solutions was based on insignificant LMR-LRT results (*p* = .338; *p* = .289), suggesting that additional latent classes were unsupported by our data. Notably, examination of individual classes revealed that the 3-class solution established three distinct trajectories, whereas the 4- and 5-class solutions primarily subdivided existing classes into smaller groups with similar trajectories.

For fathers, we similarly opted for the 3-class solution based on convergent evidence across fit indices. AIC, BIC, and saBIC values decreased markedly up to k = 3, showing little further improvement for higher class solutions. The 5-class solution for fathers was disregarded due to an insignificant LMR-LRT outcome (*p* = .403) and the lowest a posteriori probability of 0.771, which pointed to uncertain class assignments. Although the 4-class solution yielded a significant LMR-LRT result (*p* = .034), it exhibited a notable decline in the lowest a posteriori probability compared to the 3-class model (0.795 versus 0.858). The 3-class solution yielded satisfactory results in terms of entropy (0.932), as well as significant LMR-LRT and BLRT outcomes (*p* = .004; *p* < .001), and a posteriori probabilities ranging from 0.858 to 0.980. These outcomes collectively indicate strong model fit, robust class separation, and high certainty in the assignment of data points to their respective classes. Upon closer examination, similar to the findings for mothers, the 3-class solution showcased three distinct trajectories, while the 4-class solution mainly fragmented the smallest class, resulting in smaller classes with analogous trajectories.

### Growth mixture model: latent trajectory classes

**Fig. 2:**
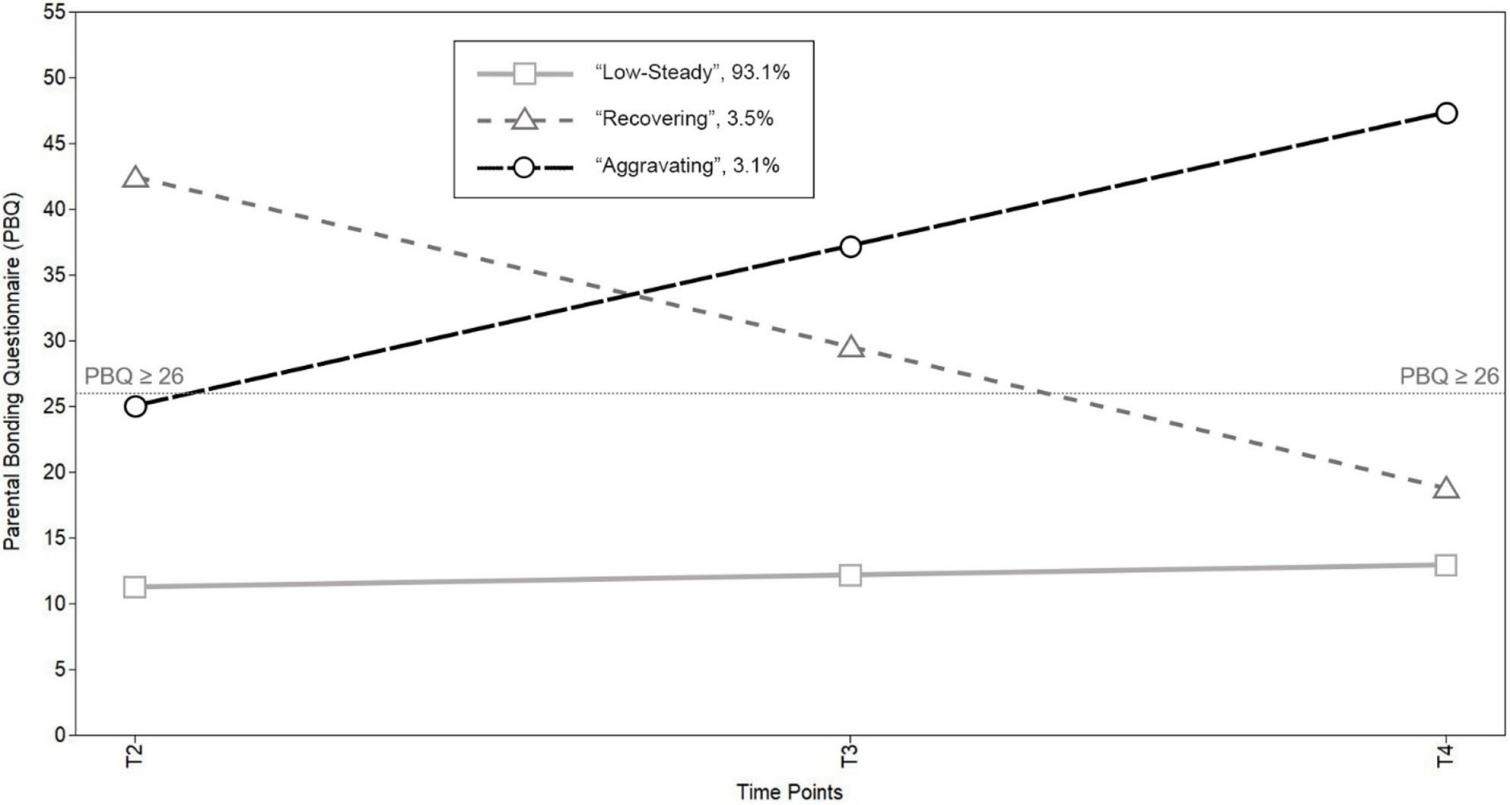
Estimated trajectory means of the 3-class solution within the maternal sample. Note. Percentages may not sum to 100% due to rounding. T2 = around eight weeks after the anticipated birth date; T3 = around 14 months after the actual birth date; T4 = around two years after the actual birth date

In both parental samples, we identified three distinct classes. For mothers, the predominant class, labeled as “low-steady”, encompassed a vast majority (93.1%, *n* = 1,640). This class exhibited a trajectory mean starting at a low and non-clinical level of bonding difficulties (intercept = 11.258 PBQ points, SE = 0.228, 95% CI [10.811, 11.705]), which remained relatively stable over the measurement period (slope = 0.765, SE = 0.090, 95% CI [0.589, 0.941]), albeit with a subtle trend towards increased bonding difficulties. The second class, denoted as “recovering”, consisted of 3.5% of mothers (*n* = 61). At T2, this class exhibited notably elevated levels of bonding difficulties (intercept = 42.469, SE = 2.924, 95% CI [36.738, 48.200]), surpassing the threshold for clinically significant bonding difficulties as defined by Brockington et al. [10]. However, over time, these levels substantially decreased (slope = -10.770, SE = 1.511, 95% CI [-13.732, -7.808]), with the trajectory mean falling below the clinical threshold between T3 and T4. The third and final class, referred to as “aggravating”, comprised 3.1% of mothers (*n* = 55). This class started just below the clinical threshold (intercept = 24.972, SE = 2.076, 95% CI [20.903, 29.041]) and exhibited a substantial subsequent increase in bonding difficulties (slope = 10.184, SE = 1.211, 95% CI [7.810, 12.558]), surpassing the clinical threshold shortly after eight weeks postpartum. The estimated trajectory means for the maternal model are depicted in Fig. 2.

**Fig. 3:**
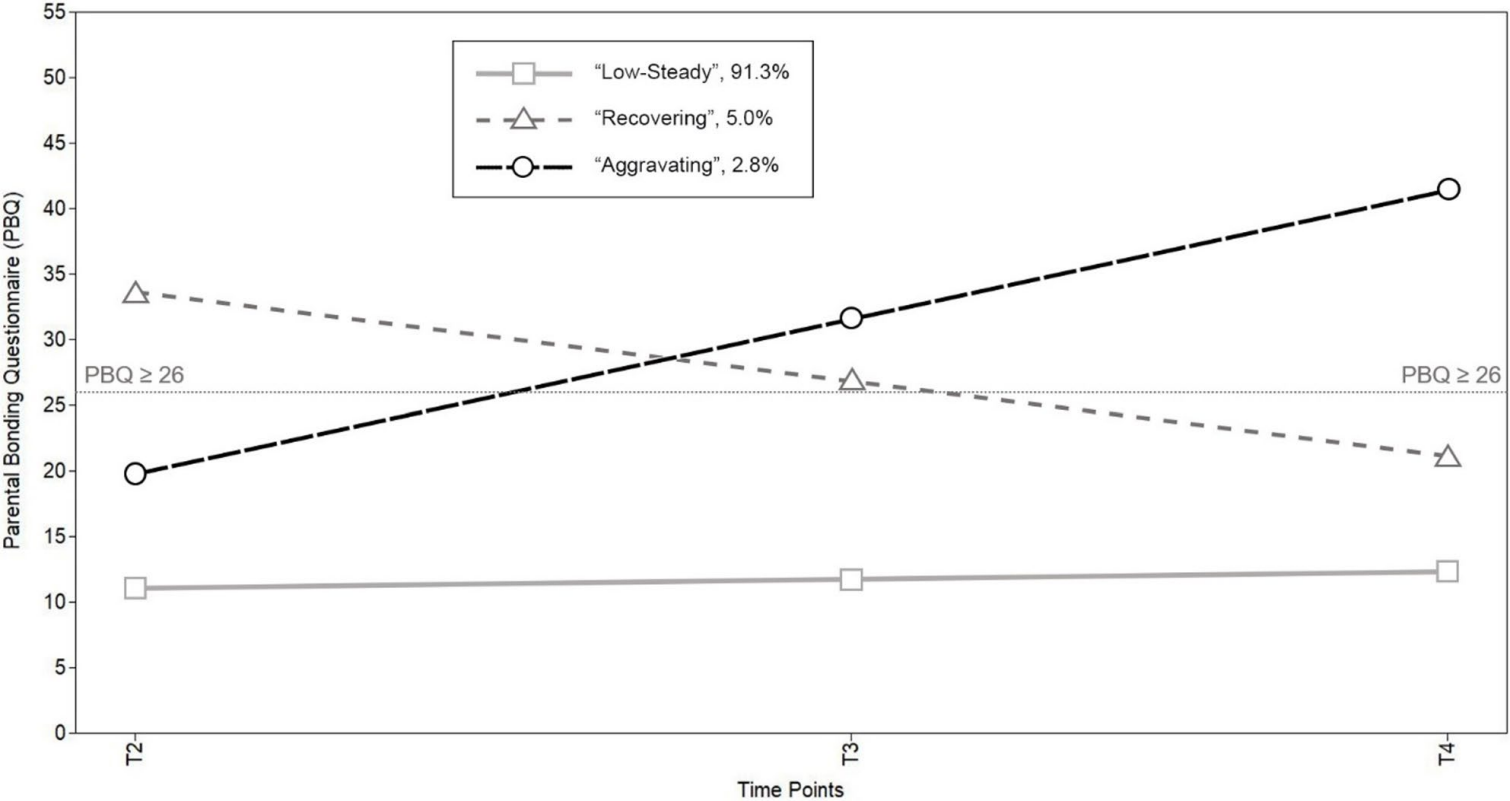
Estimated trajectory means of the 3-class solution within the paternal sample. Note. Percentages may not sum to 100% due to rounding. T2 = around eight weeks after the anticipated birth date; T3 = around 14 months after the actual birth date; T4 = around two years after the actual birth date

When examining the 3-class solution for the paternal sample, we found that the identified trajectories closely resembled those identified within the maternal sample, leading to the adoption of the same labels. The largest class identified in the paternal sample, the “low-steady” class, consisted of 91.3% of fathers (*n* = 1,030). Similar to the mothers, the estimated trajectory mean for this class started at a low level of bonding difficulties (intercept = 11.024, SE = 0.325, 95% CI [10.387, 11.661]) and remained consistent throughout the entire measurement period, with only slight increases in bonding difficulties observed over time (slope = 0.564, SE = 0.124, 95% CI [0.321, 0.807]). The second class, labeled “recovering”, included 5.0% of fathers (*n* = 56), and its trajectory mean began above the clinical threshold at T2 (intercept = 33.628, SE = 2.793, 95% CI [28.154, 39.102]). Similar to the mothers, the trajectory mean gradually decreased, heading towards non-clinical levels of bonding difficulties over time (slope = -5.636, SE = 1.452, 95% CI [-8.482, -2.790]). It fell below the threshold shortly after T3. Finally, the paternal “aggravating” class comprised 2.8% of fathers (*n* = 32). Its trajectory mean began well below the clinical threshold at T2 (intercept = 19.833, SE = 1.841, 95% CI [16.225, 23.441]). Similar to the maternal “aggravating” class, the trajectory mean for this group showed a steep increase over time, surpassing the clinical threshold between T2 and T3 (slope = 9.826, SE = 2.386, 95% CI [5.149, 14.503]). The estimated trajectory means for the paternal model are illustrated in Fig. 3.

### Growth mixture model: predictors of latent class membership

After identifying the model parameters with the best data fit for both parental samples, we conducted a multinomial logistic regression to determine predictors of class membership. The outcomes are displayed as odds ratios (ORs) in Tables 4 and 5. A concise overview of significant predictors of trajectory class membership is provided in Additional File 6 to facilitate interpretation.

**Table 4:** Multinomial logistic regression results for the maternal sample

	“Recovering” vs. “low-steady”				“Aggravating” vs. “low-steady”				“Recovering” vs. “aggravating”			
	OR	95% CI		p	OR	95% CI		p	OR	95% CI		p
		LL	UL			LL	UL			LL	UL	
Depressive symptoms	0.973	0.684	1.383	0.877	0.700	0.482	1.018	0.062	1.389	0.856	2.254	0.068
Anxiety symptoms	0.999	0.698	1.430	0.995	0.958	0.682	1.345	0.803	1.043	0.649	1.677	0.298
Somatization symptoms	0.839	0.587	1.200	0.337	1.254	0.878	1.791	0.213	0.669	0.421	1.063	0.409
Anger/hostility symptoms	1.282	0.915	1.795	0.148	1.544	1.122	2.125	0.008*	0.830	0.534	1.289	0.773
Subjective birth experience	0.471	0.339	0.656	0.000**	0.671	0.514	0.876	0.003*	0.702	0.469	1.053	0.089
Difficult child temperament	2.886	2.054	4.054	0.000**	2.162	1.431	3.266	0.000**	1.335	0.798	2.233	0.263
Relationship Satisfaction	1.008	0.753	1.350	0.958	0.834	0.595	1.168	0.290	1.209	0.799	1.831	0.493
First-time parent	0.605	0.196	1.864	0.381	0.464	0.142	1.522	0.205	1.303	0.262	6.471	0.853
Age	1.006	0.930	1.088	0.882	0.951	0.871	1.037	0.254	1.058	0.948	1.181	0.377
Education (> 10 years vs. ≤10 years)	1.405	0.558	3.539	0.471	2.454	0.578	10.422	0.224	0.573	0.111	2.952	0.533

Two sets of results were obtained for each pair of classes (i.e., “aggravating” vs. “low-steady” and “low-steady” vs. “aggravating”), yielding identical findings but with ORs in opposite directions. To prevent redundancy, only one comparison per pair of classes is displayed. *LL* Lower limit, *UL* Upper limit

* *p* < .01 (2-tailed). ** *p* < .001 (2-tailed)

**Table 5:** Multinomial logistic regression results for the paternal sample

	“Recovering” vs. “low-steady”				“Aggravating” vs. “low-steady”				“Recovering” vs. “aggravating”			
	OR	95% CI		p	OR	95% CI		p	OR	95% CI		p
		LL	UL			LL	UL			LL	UL	
Depressive symptoms	1.454	0.871	2.426	0.152	1.199	0.748	1.920	0.451	1.213	0.649	2.268	0.546
Anxiety symptoms	0.775	0.441	1.362	0.375	0.756	0.499	1.146	0.188	1.025	0.532	1.972	0.942
Somatization symptoms	0.735	0.397	1.358	0.325	1.224	0.793	1.522	0.361	0.600	0.305	1.181	0.139
Anger/hostility symptoms	1.029	0.612	1.731	0.913	1.089	0.779	1.522	0.619	0.945	0.559	1.598	0.834
Subjective birth experience	0.388	0.258	0.583	0.000**	0.669	0.477	0.940	0.021*	0.580	0.367	0.917	0.020*
Difficult child temperament	4.361	2.666	7.133	0.000**	1.859	1.114	3.102	0.018*	2.345	1.195	4.604	0.013*
Relationship Satisfaction	0.907	0.598	1.377	0.647	0.904	0.582	1.402	0.651	1.004	0.573	1.758	0.989
First-time parent	4.534	1.484	13.854	0.008*	2.124	0.662	6.819	0.206	2.135	0.484	9.404	0.316
Age	0.900	0.822	0.985	0.023*	0.891	0.814	0.974	0.011*	1.010	0.901	1.133	0.859

Two sets of results were obtained for each pair of classes (i.e., “aggravating” vs. “low-steady” and “low-steady” vs. “aggravating”), yielding identical findings but with ORs in opposite directions. To prevent redundancy, only one comparison per pair of classes is displayed. *LL *Lower limit, *UL* Upper limit

* *p* < .01 (2-tailed). ** *p* < .001 (2-tailed)

When comparing the “recovering” to the “low-steady” class in the maternal 3-class solution, a more positive subjective birth experience predicted a 52.9% lower likelihood of being assigned to the “recovering” class compared to the “low-steady” class (OR = 0.471, SE = 0.081, 95% CI [0.339, 0.656]), suggesting that mothers in the “recovering” class reported a less positive birth experience. Mothers reporting a more difficult child temperament were also more than 2.5 times as likely to be assigned to the “recovering” class (188.6% increase in odds; OR = 2.886, SE = 0.510, 95% CI [2.054, 4.054]).

Comparing the “aggravating” to the “low-steady” class, elevated anger/hostility symptoms increased the likelihood of being assigned to the “aggravating” class rather than the “low-steady” class by 54.4% (OR = 1.544, SE = 0.256, 95% CI [1.122, 2.125]). Mothers who reported a less positive subjective birth experience were 32.9% more likely to be assigned to the “aggravating” class than to the “low-steady” class (OR = 0.671, SE = 0.092, 95% CI [0.514, 0.876]). Moreover, those reporting a more difficult child temperament were more than twice as likely to be assigned to the “aggravating” class (116.2% increase in odds; OR = 2.162, SE = 0.468, 95% CI [1.431, 3.266]).

In the analysis of the paternal sample’s 3-class solution, we observed several significant associations. When comparing fathers in the “recovering” class to those in the “low-steady” class, fathers who reported a more positive subjective birth experience were 61.2% less likely to be assigned to the “low-steady” class (OR = 0.388, SE = 0.083, 95% CI [0.258, 0.583]), suggesting that those in the “recovering” class had a less positive subjective birth experience. They were also 4 times as likely to report a more difficult child temperament (OR = 4.361, SE = 1.140, 95% CI [2.666, 7.133]). Fathers with preexisting children were roughly four times more likely to belong to the “recovering” class than the “low-steady” class (353.4% increase in odds compared to first-time fathers; OR = 4.534, SE = 3.156, 95% CI [1.484, 13.854]). Additionally, younger fathers were more likely to belong to the “recovering” class than to the “low-steady” class compared to older fathers, with each additional year of age decreasing the likelihood of being assigned to the “recovering” class by approximately 10% (OR = 0.900, SE = 0.042, 95% CI [0.822, 0.985]).

Comparing fathers in the “aggravating” class to those in the “low-steady” class, those who reported a more positive subjective birth experience were 33.1% less likely to be assigned to the “aggravating” class (OR = 0.669, SE = 0.118, 95% CI [0.477, 0.940]), suggesting that fathers in the “aggravating” class experienced the birth of their child as less positive. They were also 85.9% more likely to report a more difficult child temperament than those in the “low-steady” class (OR = 1.859, SE = 0.507, 95% CI [1.114, 3.102]). Similar to the “recovering” class, younger fathers were more likely to be assigned to the “aggravating” class than to the “low-steady” class, with the odds of belonging to the “aggravating” class decreasing by 10.9% per year (OR = 0.891, SE = 0.041, 95% CI [0.814, 0.974]).

Finally, in the comparison between the “recovering” and “aggravating” classes, a more positive subjective birth experience predicted a 42% lower likelihood of being assigned to the “recovering” class (OR = 0.580, SE = 0.140, 95% CI [0.367, 0.917]), indicating that those in the “recovering” class experienced childbirth as less positive. Additionally, fathers who reported a more difficult child temperament were more likely to belong to the “recovering” class (134,5% increase in odds; OR = 2.345, SE = 0.870, 95% CI [1.195, 4.604]).

### Growth mixture model: gender-wise class comparison

Having identified similar trajectory classes in both mothers and fathers regarding bonding difficulties, this prompted us to test if bonding trajectories differ between mothers and fathers. To assess whether bonding trajectories differed by gender, we compared two nested multi-group models using the Satorra-Bentler scaled chi-square test. The comparison (χ² = 18.54; df = 6; *p* < .001) indicated that the model with equality constraints fit the data significantly worse. Thus, the trajectories in bonding difficulties were not equal between mothers and fathers, although they appeared quite similar in shape. Next, we tested gender differences in the latent growth factors within each latent class to assess whether bonding difficulties varied between mothers and fathers. Mothers in the “low-steady” class exhibited a slightly steeper increase in bonding difficulties over time compared to fathers in the corresponding class (estimated slope difference: 0.264, SE = 0.134, 95% CI [0.001, 0.527], *p* = .049). Conversely, mothers in the “recovering” class showed a significantly higher decrease in bonding difficulties over time compared to the fathers in the “recovering” class (estimated slope difference: -3.888, SE = 1.831, 95% CI [-7.477, -0.299], *p* = .034).

## Discussion

This study aimed to examine the trajectories of maternal and paternal bonding across three time points: eight weeks, 14 months, and two years postpartum. We identified distinct subgroups among mothers and fathers, each following specific bonding trajectories, dependent on the degree of bonding difficulties encountered by the parents. Furthermore, we found that specific predictor variables differentially determined the assignment of mothers and fathers to their respective trajectory classes. In the following sections, we first describe the identified trajectories of bonding difficulties for mothers and fathers, highlighting common patterns and situating the results within the current research. We then examine the predictors associated with assignment to these trajectories, emphasizing similarities and differences in effects between mothers and fathers.

While prior research suggested that parental bonding remains stable over time [13–15], no study to date has applied GMM to investigate this process. De Cock et al. [14] provided an important first step toward identifying subgroups of parents with differing bonding patterns using latent class analysis (LCA). However, because their analysis captured differences in mean levels rather than individual trajectories of change, the identified classes reflected relatively stable bonding patterns across time instead of distinct trajectories. The present study builds on and extends this work by examining a large community-based sample, adding an additional postpartum measurement point, improving parameter estimation, and incorporating various potential predictor variables, such as parental mental health factors, subjective birth experience, difficult child temperament, interpersonal factors, and sociodemographic characteristics. By applying GMM, our findings indicate that parental bonding trajectories are not uniformly stable. This approach revealed three distinct classes within our parental samples which exhibited similar patterns for both mothers and fathers. In both samples, we observed a predominant “low-steady” class with consistently low bonding difficulties throughout the first two years postpartum. There were also “recovering” classes in both samples, initially above the clinical threshold but improving over time, and “aggravating” classes that started below the threshold but worsened progressively over time. Additionally, we found various significant predictors of trajectory class membership.

Our findings partly supported the prevailing understanding of bonding development during the postpartum period as a consistently stable phenomenon. Within both the maternal and paternal samples, the “low-steady” class comprised over 90% of parents in our study. This class displayed a persistent pattern of steady and mostly low levels of bonding difficulties throughout the study period. These observations harmonize with numerous studies investigating bonding in mothers and/or fathers [13–16]. However, in contrast to prior research, our study also identified two distinct classes within both the maternal and paternal samples displaying non-steady trajectories of bonding difficulties. This suggests that, while bonding may generally exhibit stability over time for the majority of parents, a subgroup of mothers and fathers experiences a non-steady bonding development over time, which may be easily overlooked.

Interestingly, the two non-steady classes, labeled “recovering” and “aggravating”, also manifested opposing trajectories. Parents in the “recovering” class initially grappled with a notable degree of bonding difficulties following childbirth, but these challenges gradually subsided over time to the extent that the levels reached at two years postpartum were no longer considered clinically significant. In contrast, parents in the “aggravating” class started with minor issues but experienced a continuous increase in bonding difficulties over time, reaching clinically significant levels before 14 months postpartum.

From a theoretical perspective, the identification of “recovering” and “aggravating” trajectories refines prevailing assumptions about bonding stability during the postpartum period. While bonding has often been conceptualized as relatively stable once established, our findings suggest that, for a subgroup of parents, bonding represents a dynamic process that can either improve or deteriorate across the first two years. This challenges the notion that early postpartum bonding levels necessarily set a fixed developmental course and instead supports a more plastic understanding of bonding as sensitive to ongoing contextual and relational influences. In particular, the existence of a “recovering” trajectory indicates that early bonding difficulties do not inevitably persist, whereas the “aggravating” trajectory suggests that bonding problems may also emerge or intensify beyond the immediate postpartum phase.

To better understand potential gender differences in bonding difficulties, we next examined whether the identified trajectory patterns differed between mothers and fathers in both shape and levels of bonding difficulties over time.

### Gender-wise class comparison

After identifying similar bonding difficulty trajectories for both mothers and fathers, we tested whether these trajectories differed by gender. Comparing two nested multi-group models revealed gender differences in bonding difficulties despite similar trajectory shapes. Further analysis showed that mothers in the “low-steady” class exhibited a steeper increase in bonding difficulties, while mothers in the “recovering” class showed a significantly greater decrease in difficulties compared to fathers.

These differences between the corresponding “recovering” classes suggest a more profound recovery from clinical bonding difficulties over time in mothers. While all parents in the “recovering” classes faced a more challenging start, mothers made quicker progress in resolving their bonding difficulties, ultimately reaching non-clinical bonding levels by two years postpartum. These differences could be attributed to various factors, including variances in how mothers and fathers perceive, cope with, or respond to bonding difficulties, given distinctions in parental roles and parent-specific behaviors between the genders [27, 28, 147]. Since mothers traditionally take on the primary caregiving role for newborns, fathers in the “recovering” class may require additional time to adjust to the postpartum period, as they might have limited opportunities to establish close contact with their infants and participate in early childcare tasks such as feeding and soothing [28, 148]. Additionally, some fathers may lack confidence in their ability to care for an infant, potentially leading to hesitance in early parent-child interactions [148]. In contrast, mothers may find it continuously more manageable to overcome their initial bonding difficulties, given their greater involvement in childcare, reflected in the steeper decline in bonding difficulties.

Overall, mothers and fathers showed broadly similar bonding patterns, though mothers’ bonding difficulties tended to change more markedly over time – declining faster in the “recovering” class and increasing slightly more in the “low-stable” class, likely reflecting differences in caregiving roles and early parent–infant interaction opportunities.

### Predictors of trajectory class membership

By employing multinomial logistic regression, we examined which parental and child-related factors were associated with trajectory class membership. This approach allowed us to discern significant distinctions between the “low-steady” trajectory class and the clinically significant bonding trajectory classes, namely “recovering” and “aggravating”, as well as among the clinical classes themselves. While some predictors showed similar effects across mothers and fathers, others revealed important gender-specific differences.

The following section begins with predictors that showed similar effects for both mothers and fathers, before addressing gender-specific patterns. In particular, subjective birth experience and difficult child temperament emerged as shared predictors of bonding trajectories, showing comparable associations across maternal and paternal samples.

Mothers who had a negative birth experience were more often found in the “recovering” and “aggravating” classes compared to the “low-steady” class. This finding suggests that mothers may exhibit diverse coping mechanisms in relation to their mother-child bond. Some mothers who initially experience a negative birth may appear to fare reasonably well in the early stages. However, it is worth noting that a negative birth experience can potentially lead to enduring mental health issues like childbirth-related posttraumatic stress disorder symptoms [83, 84, 149]. As such, the strain on the mother-child bond might accumulate over time, ultimately resulting in the bonding patterns represented by the “aggravating” class. On the contrary, coping with a negative birth experience might be especially challenging during the early postpartum months. This is because mothers may find it difficult to process their experiences while dealing with elevated stress during the transition from pregnancy to the postpartum period [70, 96–98]. Mothers who grapple with high levels of bonding difficulties, especially in the immediate post-birth period, may, however, adapt and cope over time as they acclimatize to their new living situation. Furthermore, the influence of subjective birth experience on bonding may change over time, as affected mothers adapt to and cope with their negative birth experiences [150, 151],

A comparable pattern emerged for fathers. Specifically, a less positive birth experience increased the likelihood of fathers being assigned to the “recovering” and “aggravating” classes rather than the “low-steady” class. These findings complement the results of Seefeld et al. [80], emphasizing that the subjective birth experience can impact not only maternal, but paternal bonding as well. However, they also suggest the presence of diverse coping mechanisms that could shape how the subjective birth experience impacts the trajectory of paternal bonding.

In our comparison between fathers in the “recovering” and “aggravating” classes, it became evident that fathers in the “recovering” class reported particularly negative birth experiences, indicating an initially challenging bonding process that improved over time. This finding underscores that a challenging start characterized by a negative birth experience may initially pose strains on the father-child bonding experience. However, it also indicates the potential for improvement over time, highlighting fathers’ capacity to adapt and develop more positive bonding relationships with their children as they process negative birth experiences. Remarkably, fathers in both the “aggravating” and “recovering” classes encountered more negative birth experiences than those in the “low-steady” class. This highlights the complexity of factors influencing bonding trajectories in fathers, emphasizing that initial conditions do not predetermine persistent bonding difficulties.

Similarly, both mothers and fathers of children perceived as having a difficult temperament were more likely to belong to the clinical trajectory classes (“recovering” or “aggravating”) than to the “low-steady” class. This finding suggests the existence of diverse mechanisms when it comes to the development of maternal bonding over time. Some parents who initially experience challenges due to their child’s difficult temperament may still fare reasonably well during the early stages. However, it is worth noting that a child’s temperament tends to have a long-term impact on parental bonding [14, 94, 95]. Consequently, the strain on parental bonding might accumulate over time, ultimately leading to bonding patterns represented by the “aggravating” class. The early postpartum period, marked by sleep deprivation and the transitional phase from pregnancy to parenthood, can make a difficult child temperament even more overwhelming, exacerbating the stress experienced during this phase [70, 96–98]. However, it is important to consider that a parent’s perception of their child’s temperament could be influenced by temporary factors such as infant colic, which typically leads to increased crying during the early postpartum months [152, 153]. This factor might affect bonding differently across parents, potentially explaining why parents in the “recovering” classes were able to overcome bonding difficulties despite initially reporting a difficult temperament.

For fathers, the influence of a difficult child temperament showed a comparable yet even stronger pattern. The effect of difficult child temperament was markedly stronger for fathers than for mothers – more than 50% higher when comparing the “recovering” to the “low-steady” class. The substantial difference in estimated effects between mothers and fathers suggests that the effect of difficult child temperament on fathers’ assignment to the “recovering” class is notably stronger than its effect on mothers. This finding underscores the potential variations in how mothers and fathers perceive and respond to the challenges posed by their child’s difficult temperament, highlighting the need for further exploration of gender-specific factors influencing parental bonding trajectories.

The comparison between fathers in the “recovering” and “aggravating” classes indicated that fathers in the “recovering” class reported a more difficult child temperament. This implies that fathers who grappled with a difficult child temperament were initially prone to clinically significant bonding difficulties shortly after birth. However, these bonding difficulties exhibited signs of improvement over time, potentially signaling a change in how the child’s temperament influenced paternal bonding. A father’s perception of a difficult child temperament at eight weeks postpartum may be specifically due to frequent crying during the first weeks postpartum, often associated with infant colic. Therefore, the commonly rapid reduction in crying after eight weeks postpartum [152, 153] may result in fathers perceiving their child’s temperament as less difficult. This, in turn, could reduce the adverse impact of a difficult child temperament on paternal bonding. While the fathers in the “recovering” class reported a more difficult child temperament at eight weeks postpartum, fathers in both the “aggravating” and “recovering” classes coped with more difficult child temperaments than those in the “low-steady” class. This highlights the complexity of the relationship between paternal bonding and a difficult child temperament.

Although we observed robust associations between parental reports of a difficult child temperament and membership in clinical trajectory classes, it is essential to consider the bidirectional nature of the relationship between parental bonding and child temperament. While some studies have examined the concurrent relationship between difficult child temperament and parental bonding [45, 92] or explored how a challenging child temperament prospectively affects maternal bonding [93], others suggest that impaired parental bonding can influence how parents perceive their child’s temperament [14, 94, 95]. The significant link between a difficult child temperament and clinical trajectory classes may indeed result from the detrimental influence of a difficult child temperament on the development of parental bonding. Simultaneously, it is possible that parents in clinical trajectory classes perceive their children’s temperament as more difficult because of the bonding difficulties they experience.

When examining how parental mental health during pregnancy related to trajectory class membership, we observed that only anger/hostility symptoms significantly affected maternal bonding, while no such effect was found for paternal bonding.

We observed that mothers who exhibited greater levels of anger/hostility symptoms during pregnancy were more likely to belong to the “aggravating” class compared to the “low-steady” class. This implies that mothers with higher levels of anger/hostility symptoms were predisposed to experiencing sub-clinical bonding difficulties shortly after childbirth, which increased over time, culminating in clinically significant bonding difficulties. These findings indicate that anger/hostility symptoms during pregnancy may persist into the postpartum period and progressively impede the development of maternal bonding. Mothers experiencing elevated levels of anger/hostility symptoms may struggle to cope with the demands of caring for a newborn due to difficulties in managing emotions and stressful situations [72, 75], which could lead to worsening bonding difficulties. Additionally, a negative feedback loop between maternal anger/hostility symptoms and infant responses could intensify these challenges.

Among fathers, sociodemographic factors – specifically first-time parenthood and age – were associated with bonding trajectories. First-time fathers were about four times less likely to be assigned to the “recovering” class compared to the “low-steady” class. Having preexisting children was therefore associated with higher levels of bonding difficulties shortly after childbirth, but these difficulties improved over time. This phenomenon may be attributed to fathers with multiple children having to navigate the demands of multiple offspring during the demanding postpartum phase, when the entire family is adjusting to new circumstances. Mothers usually take on the primary caregiving role for the newborn, enabling fathers to offer crucial attention and support to older siblings, who may exhibit challenging behaviors in response to familial changes and benefit from extra care [107, 108, 110, 154]. This increased attention to older children might lead to fathers having less interaction with the newborn, resulting in initial bonding difficulties. However, as the family adjusts to the new living situation, fathers have the opportunity to form a stable bond with their youngest child over time, as represented by the decreasing trajectory of bonding difficulties in the “recovering” class. Nevertheless, while the estimated effect indicates a strong positive association, it is important to note that the wide confidence interval suggests some uncertainty in the extent of this estimate. Given the uncertainty reflected in the wide confidence interval, further research may be needed to better understand the nature and strength of this association.

Another noteworthy observation relates to the impact of age on paternal bonding. Our analysis unveiled that fathers assigned to either the “aggravating” or “recovering” classes were notably younger compared to those assigned to the “low-steady” class. This suggests that younger fathers were more likely to experience a non-stable bonding trajectory characterized by clinically significant bonding difficulties at one or more points during the study’s duration. Interestingly, these findings diverge from some prior research, which had found inconsistent associations between higher age and increased bonding difficulties [14, 16]. In essence, these results indicate that age does not consistently predict bonding difficulties in fathers. Instead, younger fathers appear to be at a higher risk of experiencing fluctuating bonding difficulties during the postpartum period.

While we identified several significant predictors of trajectory class membership in our parental samples, some variables had no discernible impact. Considering all the findings in the maternal model, we did not identify any significant impact of depression, anxiety and somatization symptoms during pregnancy, as well as relationship satisfaction, first-time parenthood, age, and education on trajectory class membership. The same holds true for depressive, anxiety, somatization, anger/hostility symptoms, and relationship satisfaction when considering the paternal model.

Overall, these analyses illustrate that the development of parental bonding over the first two years postpartum is shaped by a combination of parent- and child-related factors, with some effects being common across parents and others showing gender-specific patterns.

### Strengths and limitations

This study has several strengths that contribute to its significance. We conducted a comprehensive examination of maternal and paternal bonding trajectories during the early postpartum period, unveiling previously unrecognized patterns of bonding difficulties.

In this study, we adopted a GMM approach, which offers advantages over traditional analytical methods. GMM enables the detection of distinctive trajectories, accommodates missing data points, and reveals subtle patterns that might be overlooked using other approaches [155–157]. Furthermore, this study was the first to identify the clinical “recovering” and “aggravating” classes of bonding difficulties. Two main factors could contribute to their oversight in previous studies: methodology and sample size. First, the methodology employed in this investigation is relatively novel within the field of peripartum research, with limited prior application for analyzing parental bonding trajectories. While one study conducted by de Cock et al. [14] employed a similar approach, their method could only identify groups with comparable patterns across time points and did not estimate latent growth factors or within-person change. De Cock et al. [14] also highlighted the scarcity of studies using large population-based samples to investigate various patterns of parental bonding, especially the transitions from non-clinical bonding to clinical bonding difficulties and vice versa. In our study, we have offered initial insights into the diverse trajectories that the development of bonding can follow over time.

Additionally, the inclusion of various predictors, encompassing mental health factors, subjective birth experience, difficult child temperament, interpersonal factors, and sociodemographic characteristics enriched our understanding of the intricate dynamics of bonding. This comprehensive analysis of both maternal and paternal bonding experiences addressed a significant gap in the existing literature.

However, despite our best efforts, it is essential to recognize that our study has some limitations. With three postpartum assessment points, the GMM was constrained to linear growth factors, as the estimation of nonlinear trajectories (e.g., quadratic or cubic) requires a minimum of four measurement points. While a linear model is therefore the only statistically viable specification given the current design, future studies incorporating additional measurement points would allow for the examination of potentially nonlinear bonding trajectories. Beyond these structural constraints, while we identified several significant predictors of trajectory class membership, we did not find a significant influence of variables that have previously been recognized as predictors of parental bonding, such as anxiety symptoms and relationship satisfaction. It is essential to underline that this lack of significance does not necessarily mean that these variables have no influence on parental bonding; rather, it indicates that these factors do not distinguish between the various bonding difficulty trajectories observed in this study. These variables might still be relevant to maternal and paternal bonding in a broader sense, influencing bonding experiences differently or contributing to bonding difficulties in ways that are not captured by the trajectory classes examined here. On the same note, the exclusion of OCD symptoms from both the maternal and paternal models due to multicollinearity and sensitivity analyses precludes drawing conclusions about the role of this factor in parental bonding trajectories. Likewise, education was excluded from the paternal model for the same reason. Future research should aim to replicate these analyses in larger or more diverse samples that allow for the simultaneous inclusion of these conceptually relevant predictors to clarify their potential contribution.

Furthermore, apart from subjective birth experience and difficult child temperament, all included predictor variables were assessed during pregnancy. Their potential influence on parental bonding may undergo changes during the transition from pregnancy to the postpartum period. This highlights the complexity of the factors affecting parental bonding.

Also, the multinomial logistic regression used to identify significant predictors of trajectory class membership required complete data on the predictor variables. Consequently, some parents who otherwise met the study criteria were excluded due to missing data on the questionnaires used to measure these variables. A comparison between the final sample and the excluded participants revealed some notable differences. Excluded mothers reported higher levels of anger/hostility symptoms during pregnancy, potentially leading to an underestimation of the impact of these symptoms on trajectory class membership. Excluded fathers were also older than those included in the study, possibly overestimating the effect of age on trajectory class membership. Additionally, it is worth noting that excluded fathers exhibited higher levels of depressive symptoms compared to those in the final sample. This difference in depressive symptomatology among the excluded participants could have impaired the study’s capacity to identify an effect of depressive symptoms on trajectory class membership. Although we prioritized conceptual clarity and transparency in adopting a complete-case approach, future studies could implement multiple imputation strategies for predictor variables to further assess the robustness of class–predictor associations and mitigate potential selection bias. Given that attrition analyses revealed some significant differences between included and excluded participants, such strategies may be particularly warranted to further safeguard against potential selection bias.

Another limitation concerns the absence of certain theoretically relevant variables, such as trauma history, attachment style, and prepartum bonding, which were not included in the main DREAM questionnaire battery. Future research should aim to integrate such variables to further elucidate the mechanisms underlying different bonding trajectories. Moreover, all measures in the present study were based on self-report questionnaires, which, although enabling efficient data collection in a large cohort, may introduce shared method variance and social desirability bias. This is particularly relevant for the association between parental bonding and difficult child temperament, where shared method variance may partially account for the observed associations, and the design precludes conclusions about the direction of this relationship. While observational assessments of parent–infant interaction provide valuable complementary information, parental bonding has been conceptualized as parents’ connection to their child, encompassing affective, behavioral, cognitive, and underlying neurobiological components, and unfolding as a developmental process that begins with the intention to conceive and continues throughout infancy [11]. While self-report questionnaires primarily capture parents’ internal experiences and representations of this relationship, observational approaches focus on externally observable interaction patterns within specific caregiving contexts. Empirical findings suggest that associations between self-reported bonding and observed interaction quality are modest, indicating that these methods tap into related but partially distinct facets of the early parent–child relationship [116, 158]. Future research would therefore benefit from integrating multimethod approaches – including (dyadic) observational assessments – not only to reduce potential bias and enhance construct validity, but also to examine whether the trajectory classes identified through self-reported bonding correspond to parallel developmental patterns in observed interaction over time.

While we conducted a thorough analysis of parental bonding trajectories in a large community-based sample, it is important to note that community-based samples typically include a limited number of participants with clinically significant symptoms. As a result, non-clinical bonding cases were more common in our sample, leading to a notable difference in group size between the non-clinical “low-steady” and the clinical “recovering” and “aggravating” classes. The smaller sample size of the clinical trajectory classes may have resulted in less precise estimates of the impact of predictor variables on bonding difficulties, as smaller groups increase the likelihood of Type II errors and reduce statistical power [159, 160]. In particular, some regression estimates for the smaller “recovering” and “aggravating” classes should be interpreted with caution, as small class sizes may contribute to standard error inflation and, consequently, to the magnitude of observed odds ratios, which should therefore be considered alongside their confidence intervals. To address this issue, further research could concentrate on trajectory classes within clinical samples, enabling a more comprehensive analysis of parents struggling with clinical bonding difficulties.

Furthermore, our sample was characterized by relatively high levels of education, as extensively discussed in the study protocol [113]. Also, the study exclusively focused on parents in committed and opposite-sex relationships. Consequently, our findings may not be readily generalizable to families with lower educational backgrounds or alternative family structures, such as single-parent households, blended families (i.e. with one or both parents in a relationship having children from previous relationships), and LGBTQ+ families. Additionally, some parents encountered clinical bonding difficulties according to their sum scores at various points during the study but were still assigned to the “low-steady” classes. This could suggest that, while these parents faced bonding challenges, their overall trajectory remained more stable over time compared to those in the “aggravating” or “recovering” classes. This limitation highlights the possibility that the “low-steady” class may not fully capture the complexity or temporality of bonding difficulties, as individuals in a GMM are grouped based on similarities, and this classification is probabilistic in nature. This may lead to some individuals being misclassified and their bonding challenges not being fully represented.

Finally, the original version of the SCL-90-R has been discussed in prior research regarding its factorial structure, which has shown some variability across studies [161–163]. This is particularly evident in samples with lower levels of psychological distress [164], which is commonly observed in non-clinical samples. While the SCL-90-R serves as a useful screening tool, it is designed to complement rather than replace comprehensive clinical assessments. Recognizing these considerations is important, as it highlights how variations in the tool’s structural consistency might affect the interpretation of parental mental health and bonding difficulties, particularly in non-clinical samples where its sensitivity could be more limited.

### Implications for research and clinical applications

Our study extends prior research by uncovering previously undetected trajectory classes, challenging the notion of bonding as a uniformly stable phenomenon. Instead of assuming that bonding is always consistent, it is important for researchers and clinicians to understand that there can be various patterns of bonding difficulties. This understanding calls for customized interventions for parents who are at risk of experiencing unstable bonding, based on the predictors of their bonding patterns. While the observational design of this study limits direct causal inference, the prospective assessment of predictors across multiple timepoints provides meaningful directional support for the following implications, which we offer as evidence-informed guidance for clinical practice and future research.

In this study, we identified non-steady clinical trajectory classes of parental bonding during the first two years after childbirth. While we explored a wide range of potential predictors, further research is essential to gain a deeper understanding of these clinical classes. Future studies should expand the investigation of parental bonding trajectories, extending beyond the postpartum period, to determine whether these non-steady patterns go on to fluctuate or stabilize over time. It is also crucial to continue examining the influence of potential predictors on trajectory class membership following childbirth, including the factors assessed during pregnancy in this study. Additionally, considering variables related to work and employment could provide valuable insights. Although Schaber et al. [165] demonstrated that the duration of parental leave did not impact paternal bonding after accounting for relevant factors, it remains valuable to consider the broader context of work-related variables, as there may be other contextual or nuanced aspects of employment that influence bonding in ways not yet fully explored.

To address the challenge of relatively small clinical trajectory classes in community samples, future research could focus on assessing bonding difficulties within larger clinical samples. A larger clinical sample would enable more precise estimation of the impact of predictor variables on bonding difficulties, leading to a more refined understanding of the factors influencing clinical trajectory classes. Moreover, further exploration of the “recovering” trajectory classes could yield valuable insights into the dynamics of recovery from bonding difficulties. Additionally, it is essential to delve deeper into the “aggravating” trajectories, as their long-term course suggests a potential chronicity of bonding difficulties.

Future research should investigate the impact of these non-steady clinical bonding trajectories on parental mental health during the postpartum period and beyond, an aspect that was not within the scope of this study. While many studies have explored how parental mental health can affect bonding, there is a limited body of research on how postpartum bonding difficulties might influence parental mental health in the future. However, de Cock et al. [1] found a prospective link between parental bonding difficulties and perceived parenting stress, suggesting that bonding difficulties could have a long-term impact on parental mental health.

Additionally, it is crucial to gain a deeper understanding of how these clinical bonding trajectories may influence the development and mental health of children. Several studies have already established a significant link between maternal bonding and a child’s behavioral and socio-emotional development [1–4]. Furthermore, numerous studies on neurodevelopment emphasize the first two years postpartum as a uniquely sensitive period for child development [166–169]. Considering parents in the “recovering” classes experience a gradual reduction in bonding difficulties over time, potentially mitigating the initial impact on their children, it is essential to recognize that those in the “aggravating” classes face persistent worsening of bonding difficulties, which could have enduring consequences for child development and mental health. These children may encounter challenges across various domains, with potential differences depending on whether their parents follow a “recovering” or “aggravating” trajectory of bonding difficulties. Conducting child-centered research on the effects of these non-steady bonding trajectories can shed light on the specific difficulties these children may encounter and help tailor interventions to their unique needs.

Since mothers typically assume the primary caregiving role for newborns, fathers may need additional time to establish a stable bond with their children. To promote paternal bonding, fathers may benefit from educational initiatives that teach safe methods for interacting with their newborns, including techniques like baby massage and early bottle-feeding [147, 170, 171]. These educational programs could be integrated into standard postpartum care, similar to breastfeeding support provided to mothers [172].

This study also uncovered an interesting link between maternal anger/hostility symptoms and bonding difficulties, indicating that elevated anger/hostility symptom levels may hinder maternal bonding and potentially intensify these difficulties over time. These results underscore the significance of early identification and support for expectant mothers with high levels of anger/hostility symptoms, with interventions aimed at reducing anger/hostility symptoms and enhancing coping skills to potentially improve the trajectory of maternal bonding.

It has become evident that both mothers and fathers reporting negative birth experiences are susceptible to bonding difficulties. Therefore, healthcare professionals should consider the impact of birth experiences on parent-child bonding for both parents and offer appropriate support and guidance. To mitigate the potential impact of a negative birth experience on parental bonding, interventions should be directed toward parents both before and after childbirth. Promoting feelings of control, choice, and respect during childbirth can contribute to a more positive birth experience in mothers [173–177]. Targeted information materials and counseling can also assist parents in coping with negative birth experiences [173, 178–181]. This would require a structured assessment of the subjective birth experiences in both parents to promptly identify those affected by a negative birth experience [173]. Midwives can play a crucial role in providing support and counselling [173, 179–181], targeting both parents, if possible, and suggesting psychotherapeutic counselling if needed. However, since the fathers’ perspective on birth experiences has received considerably less attention in both research and practice compared to mothers, further research is necessary to determine how to effectively support fathers during and after childbirth to promote a more positive subjective birth experience.

In this study, another significant predictor of trajectory class membership in fathers was first-time parenthood. Being a father to preexisting children was linked to a “recovering” trajectory of bonding difficulties, possibly reflecting a delayed development of paternal bonding towards the newborn due to increased childcare responsibilities for older siblings during this period [107, 108, 110, 154]. To alleviate the load on these fathers, interventions should take into account the needs of older siblings during the postpartum transition. While the reactions of older siblings to a new baby can vary, those facing the most challenges with adjustment may particularly benefit from tailored support programs [110]. Although the interventions did not specifically target siblings after the arrival of a newborn, studies have shown that emotional regulation training for 4- to 8-year-old siblings improved not only the children’s regulatory skills but also their parents’ emotional regulation abilities [182, 183]. Thus, providing support for siblings can help alleviate the stress of the transitional period after the arrival of a new sibling for both fathers and older siblings, potentially creating a more conducive environment for the development of paternal bonding with the newborn child.

Analyzing the fathers in our study also revealed that younger fathers were more susceptible to being part of the clinical trajectory classes identified in this research, indicating an elevated vulnerability to clinical bonding difficulties. Implementing educational and support programs tailored for younger fathers has demonstrated its efficacy in improving their adaptation to the demands of newborn care [184, 185], fostering more positive bonding experiences. Nonetheless, many programs aimed at supporting young parents struggle to effectively involve fathers [186, 187]. Providing widely accessible and engaging programs to young fathers shortly after childbirth may effectively assist them in overcoming postpartum bonding difficulties.

Finally, parental education programs that incorporate mindfulness and mentalization have demonstrated promising outcomes, including reduced symptoms of maternal mental health issues, enhanced mother-child relationships, and improved co-parenting [188–192]. Parental education programs should ideally target both mothers and fathers [193, 194] and include information about potential challenges during the postpartum period, such as bonding difficulties and their contributing factors. Some aspects, such as addressing peripartum depression in mothers, are already integrated into certain parental education programs. However, other factors such as the subjective birth experience and managing a challenging child temperament should also be incorporated into these programs, given their recognized importance in parental bonding. Recent initiatives are beginning to recognize the significance of these factors [195, 196]. Providing parents with insights and effective coping mechanisms could significantly contribute to preventing or mitigating bonding difficulties.

## Conclusion

This study delved into the longitudinal trajectories of maternal and paternal bonding during the first two years of the postpartum period, identifying distinct classes representing different courses of bonding difficulties over time. Within both the maternal and paternal samples, we observed three distinct trajectories: “low-steady”, “recovering”, and “aggravating”, effectively challenging the notion of bonding as a uniformly stable phenomenon, facilitated by the application of Growth Mixture Modeling. We identified several significant predictors of trajectory class membership. For mothers, predictors included anger/hostility symptoms, subjective birth experience, and difficult child temperament. In contrast, fathers’ trajectory class membership was predicted by subjective birth experience, difficult child temperament, first-time parenthood, and age. Our findings emphasize the importance of acknowledging the possibility of diverse, non-steady bonding patterns, highlighting the necessity for customized interventions when necessary. This study’s insights on parental bonding have led to several important recommendations for supporting parents at risk of experiencing non-steady clinical bonding trajectories. Furthermore, the scarcity of research regarding the paternal perspective on various factors surrounding childbirth and parenthood has been highlighted, along with potential directions for further exploration in future studies concerning clinical bonding trajectories.

## Supplementary information

Supplementary Material 1.

Supplementary Material 2.

Supplementary Material 3.

Supplementary Material 4.

Supplementary Material 5.

Supplementary Material 6.

Supplementary Material 7.

## Data Availability

The dataset analyzed during the current study is not publicly available due to ethical and legal restrictions, as the informed consent did not include permission for public data sharing. The datasets used and analyzed during the current study are available from the principal investigator on reasonable request. Requests should be directed to susan.garthus-niegel@ukdd.de.

## Abbreviations

AIC: Akaike Information Criterion
BIC: Bayesian Information Criterion
BLRT: Bootstrap Likelihood Ratio Test
EPDS: Edinburgh Postnatal Depression Scale
FIML: Full-Information Maximum-Likelihood
GMM: Growth Mixture Model
ICQ: Infant Characteristic Questionnaire
LMR-LRT: Lo, Mendell, and Rubin Likelihood Ratio Test
MD: Mahalanobis distance
MI: Multiple Imputation
OCD: Obsessive-Compulsive Disorder
OR: odds ratio
PBQ: Postpartum Bonding Questionnaire
PFB-K: short version of the Partnership Questionnaire
saBIC: Sample-Size Adjusted Bayesian Information Criterion
SCL-90-R: Symptom Checklist-90-Revised
SIL: Salmon’s Item List
