# Supplementary material for: Understanding parental bonding in the first two years after birth: exploring family predictors using growth mixture modeling
Source: BMC Psychol. 2026 May 27;14:784. doi: 10.1186/s40359-026-04788-9 (PMC13214287; doi:10.1186/s40359-026-04788-9)
Supplement: Supplementary file 1 — Supplementary Material 1. [file 40359_2026_4788_MOESM1_ESM.docx]

# Multicollinearity Assessment and Sensitivity Analyses

This file provides supplementary methodological details and additional analyses. References specific to this file are listed at the end.

To assess potential multicollinearity among predictors prior to the main analyses, we computed pairwise Pearson correlation coefficients between all continuous predictor variables for the maternal and paternal samples, respectively (see Tables 1 and 2). Correlation coefficients exceeding |r| = .7 are commonly considered indicative of problematic multicollinearity (1). Although no correlations in our data reached this threshold, several predictors – particularly depressive, anxiety, obsessive–compulsive (OCD), and somatization symptoms – showed moderate intercorrelations above |r| = .5 in both parental samples. These associations are theoretically expected, as the SCL-90-R subscales measure overlapping aspects of general psychological distress and are known to be moderately to highly interrelated (2–5).

To detemine whether such overlap might bias regression estimates through suppression or redundancy effects, we re-estimated all models by testing each potentially collinear predictor separately. The results were largely consistent with the main analyses but revealed several meaningful changes, summarized in Tables 3 and 4.

In the maternal sample, these differences appeared in the comparison of the “aggravating” versus “low-steady” class. When tested individually, the previously counterintuitive finding that mothers in the “aggravating” class reported fewer depressive symptoms than those in the “low-steady” class was no longer significant, and the odds ratio reversed direction. Concurrently, OCD symptoms became a significant predictor of class membership. This pattern suggested suppression effects caused by shared variance among correlated mental health variables. Consequently, OCD symptoms were excluded from the final maternal model to enhance interpretive clarity and reduce redundancy.

For fathers, the same approach revealed several adjustments when comparing the “recovering” to the “low-steady” class. Fathers in the “recovering” class now reported significantly higher depressive symptoms and a trend-level increase in OCD symptoms compared with the “low-steady” class, with the OCD odds ratio reversing direction relative to the original model. In addition, education and first-time parenthood were moderately correlated (r = .58). This correlation likely reflects cohort-related socioeconomic timing effects, whereby men with higher educational attainment tend to postpone fatherhood due to extended educational and career trajectories (6–9). When tested individually, the previously significant effect of first-time parenthood was no longer significant. Given the small number of fathers with less than 10 years of schooling and the resulting unstable estimates, both education and OCD symptoms were excluded from the final paternal model.

Table 1: Results of Multicollinearity Analysis in the Maternal Sample

|  | 1. | 2. | 3. | 4. | 5. | 6. | 7. | 8. | 9. | 10. | 11. |
| --- | --- | --- | --- | --- | --- | --- | --- | --- | --- | --- | --- |
| 1. Depressive symptoms | -- |  |  |  |  |  |  |  |  |  |  |
| 2. Anxiety symptoms | .561^**^ | -- |  |  |  |  |  |  |  |  |  |
| 3. Obsessive-compulsive   symptoms | .561^**^ | .642^**^ | -- |  |  |  |  |  |  |  |  |
| 4. Somatization symptoms | .411^**^ | .520^**^ | .566^**^ | -- |  |  |  |  |  |  |  |
| 5. Anger/hostility symptoms | .542^**^ | .508^**^ | .557^**^ | .430^**^ | -- |  |  |  |  |  |  |
| 6. Subjective birth   experience | -.126^**^ | -.168^**^ | -.158^**^ | -.141^**^ | -.102^**^ | -- |  |  |  |  |  |
| 7. Difficult child   temperament | .083^**^ | .077^**^ | .092^**^ | .085^**^ | .042 | -.208^**^ | -- |  |  |  |  |
| 8. Relationship satisfaction | -.204^**^ | -.085^**^ | -.190^**^ | -.152^**^ | -.250^**^ | .031 | -.050^*^ | -- |  |  |  |
| 9. First-time parent | .097^**^ | -.001 | -.002 | .022 | .114^**^ | .176^**^ | -.107^**^ | -.283^**^ | -- |  |  |
| 10. Age | -.015 | -.014 | -.076^**^ | -.071^**^ | -.052^*^ | -.026 | .011 | -.094^**^ | .306^**^ | -- |  |
| 11. Education (>10 years vs.   <=10 years) | -.101^**^ | -.055^*^ | -.055^*^ | -.091^**^ | -.056^*^ | -.065^**^ | .111^**^ | .043 | -.053^*^ | .044 | -- |

*Note.* We computed all associations utilizing the Pearson correlation coefficient. Significant correlations might indicate conceptual overlap.

** p < 0.05 (two-tailed). ** p < 0.01 (two-tailed).*

Table 2: Results of Multicollinearity Analysis in the Paternal Sample

|  | 1. | 2. | 3. | 4. | 5. | 6. | 7. | 8. | 9. | 10. | 11. |
| --- | --- | --- | --- | --- | --- | --- | --- | --- | --- | --- | --- |
| 1. Depressive symptoms | -- |  |  |  |  |  |  |  |  |  |  |
| 2. Anxiety symptoms | .596^**^ | -- |  |  |  |  |  |  |  |  |  |
| 3. Obsessive-compulsive   symptoms | .621^**^ | .649^**^ | -- |  |  |  |  |  |  |  |  |
| 4. Somatization symptoms | .453^**^ | .577^**^ | .556^**^ | -- |  |  |  |  |  |  |  |
| 5. Anger/hostility symptoms | .502^**^ | .560^**^ | .565^**^ | .386^**^ | -- |  |  |  |  |  |  |
| 6. Subjective birth   experience | -.137^**^ | -.147^**^ | -.130^**^ | -.107^**^ | -.083^**^ | -- |  |  |  |  |  |
| 7. Difficult child   temperament | .075^*^ | 0.057 | .115^**^ | .010 | .066^*^ | -.146^**^ | -- |  |  |  |  |
| 8. Relationship satisfaction | -.209^**^ | -.136^**^ | -.153^**^ | -.130^**^ | -.246^**^ | .061^*^ | -.070^*^ | -- |  |  |  |
| 9. First-time parent | .136^**^ | .063^*^ | .065^*^ | .090^**^ | .166^**^ | .098^**^ | -.032 | -.292^**^ | -- |  |  |
| 10. Age | .012 | .018 | -.032 | .031 | .017 | .031 | .010 | -.069^*^ | .336^**^ | -- |  |
| 11. Education (>10 years vs.   <=10 years) | -.096^**^ | -.069^*^ | -.007 | -.124^**^ | -.077^**^ | -.097^**^ | .098^**^ | .027 | .582^**^ | -.029 | -- |

*Note.* We computed all associations utilizing the Pearson correlation coefficient. Significant correlations might indicate conceptual overlap.

** p < 0.05 (two-tailed). ** p < 0.01 (two-tailed)*

Table 3: Multinomial logistic regression results for predictors with moderate multicollinearity, tested individually (maternal sample)

|  | “Recovering” vs. “low-steady” | | | |  | “Aggravating” vs. “low-steady” | | | |  | “Recovering” vs. “aggravating” | | | |
| --- | --- | --- | --- | --- | --- | --- | --- | --- | --- | --- | --- | --- | --- | --- |
|  | OR | 95% CI | | p |  | OR | 95% CI | | p |  | OR | 95% CI | | p |
|  |  | LL | UL |  |  |  | LL | UL |  |  |  | LL | UL |  |
| Depressive symptoms | 1.192 | 0.979 | 1.451 | .081 |  | 1.123 | 0.870 | 1.449 | .372 |  | 1.061 | 0.776 | 1.451 | .711 |
| Anxiety symptoms | 1.212 | 0.984 | 1.494 | .071 |  | 1.239 | 0.978 | 1.570 | .075 |  | 0.978 | 0.719 | 1.330 | .888 |
| Obsessive-compulsive symptoms | 0.961 | 0.730 | 1.264 | .774 |  | 1.454 | 1.188 | 1.780 | .000** |  | 0.661 | 0.562 | 0.842 | .015* |
| Somatization symptoms | 1.104 | 0.891 | 1.368 | .364 |  | 1.384 | 0.997 | 1.918 | .052 |  | 0.798 | 0.596 | 1.068 | .129 |
| Anger/hostility symptoms | 1.182 | 0.925 | 1.511 | .182 |  | 1.148 | 0.839 | 1.572 | .390 |  | 0.816 | 0.588 | 1.132 | .223 |

*Note.* Separate multinomial logistic regression models were estimated for each predictor variable that showed moderate multicollinearity in the full model (|r| > .5). Two sets of results were obtained for each pair of classes (i.e., “aggravating” vs. “low-steady” and “low-steady” vs. “aggravating”), yielding identical findings but with ORs in opposite directions. To prevent redundancy, only one comparison per pair of classes is displayed. LL = lower limit; UL = upper limit.
** p < .01 (2-tailed). ** p < .001 (2-tailed).*

Table 4: Multinomial logistic regression results for predictors with moderate multicollinearity, tested individually (paternal sample)

|  | “Recovering” vs. “low-steady” | | | |  | “Aggravating” vs. “low-steady” | | | |  | “Recovering” vs. “aggravating” | | | |
| --- | --- | --- | --- | --- | --- | --- | --- | --- | --- | --- | --- | --- | --- | --- |
|  | OR | 95% CI | | p |  | OR | 95% CI | | p |  | OR | 95% CI | | p |
|  |  | LL | UL |  |  |  | LL | UL |  |  |  | LL | UL |  |
| Depressive symptoms | 1.392 | 1.113 | 1.742 | .004* |  | 1.291 | 0.938 | 1.777 | .117 |  | 1.079 | 0.750 | 1.551 | .684 |
| Anxiety symptoms | 1.092 | 0.849 | 1.405 | .492 |  | 1.084 | 0.814 | 1.445 | .581 |  | 1.007 | 0.701 | 1.448 | .968 |
| Obsessive-compulsive symptoms | 1.257 | 0.996 | 1.558 | .054* |  | 1.210 | 0.897 | 1.633 | .213 |  | 1.039 | 0.723 | 1.492 | .836 |
| Somatization symptoms | 1.121 | 0.886 | 1.419 | .342 |  | 1.251 | 0.990 | 1.582 | .061 |  | 0.896 | 0.661 | 1.214 | .479 |
| Anger/hostility symptoms | 1.209 | 0.990 | 1.477 | .062 |  | 1.216 | 0.937 | 1.580 | .142 |  | 0.994 | 0.737 | 1.342 | .969 |
| First-time parent | 1.720 | 0.851 | 3.478 | .131 |  | 1.259 | 0.488 | 3.245 | .634 |  | 1.366 | 0.432 | 4.321 | .595 |
| Education (>10 years vs. ≤10 years) | 2.026 | 0.838 | 4.900 | .117 |  | 1.464 | 0.553 | 3.874 | .442 |  | 1.384 | 0.377 | 5.080 | .625 |

*Note.* Separate multinomial logistic regression models were estimated for each predictor variable that showed moderate multicollinearity in the full model (|r| > .5). Two sets of results were obtained for each pair of classes (i.e., “aggravating” vs. “low-steady” and “low-steady” vs. “aggravating”), yielding identical findings but with ORs in opposite directions. To prevent redundancy, only one comparison per pair of classes is displayed. LL = lower limit; UL = upper limit.
** p < .01 (2-tailed). ** p < .001 (2-tailed).*

# References

1. Dormann CF, Elith J, Bacher S, Buchmann C, Carl G, Carré G, et al. Collinearity: A review of methods to deal with it and a simulation study evaluating their performance. Ecography (Cop). 2013;36(1):27–46.

2. Baez LM, Newport DJ, Stowe ZN, Knight BT, Heller AS. The severity and role of somatic depressive symptoms in psychological networks in a longitudinal sample of peripartum women. J Psychiatr Res. 2021;142:283–9.

3. de Bles NJ, Rius Ottenheim N, van Hemert AM, Pütz LEH, van der Does AJW, Penninx BWJH, et al. Trait anger and anger attacks in relation to depressive and anxiety disorders. J Affect Disord. 2019;259:259–65.

4. Hankin BL, Snyder HR, Gulley LD, Schweizer TH, Bijttebier P, Nelis S, et al. Understanding comorbidity among internalizing problems: Integrating latent structural models of psychopathology and risk mechanisms. Dev Psychopathol. 2016;28(4):987–1012.

5. Yap K, Mogan C, Kyrios M. Obsessive-compulsive disorder and comorbid depression: The role of OCD-related and non-specific factors. J Anxiety Disord. 2012;26(5):565–73.

6. Einiö E, Goisis A, Myrskylä M. Is the relationship between men’s age at first birth and midlife health changing? Evidence from two British cohorts. SSM - Popul Heal. 2019;8.

7. Grätz M, Wiborg ØN. Parental ages and the intergenerational transmission of education: evidence from Germany, Norway, and the United States. Eur Soc. 2024;26(5):1444–71.

8. Hershkovitz-Freudenthal A, Lavenda O. Factors associated with contemporary fatherhood. Front Psychol. 2024;15.

9. Shpiegel S, Aparicio EM, Smith R, Grinnell-Davis C, King B. Early fatherhood and socioeconomic outcomes among young men transitioning from foster care in the United States. Child Youth Serv Rev. 2022;133.
