# Supplementary material for: Understanding parental bonding in the first two years after birth: exploring family predictors using growth mixture modeling
Source: BMC Psychol. 2026 May 27;14:784. doi: 10.1186/s40359-026-04788-9 (PMC13214287; doi:10.1186/s40359-026-04788-9)
Supplement: Supplementary file 2 — Supplementary Material 2. [file 40359_2026_4788_MOESM2_ESM.docx]

# Outlier detection

To identify multivariate outliers, Mahalanobis distances (MD) were computed for the full predictor set separately for mothers and fathers, using standard linear regression models with PBQ scores at T2, T3, and T4 as dependent variables. A mean MD was then calculated for each participant to obtain a single multivariate index. To flag potentially multivariate outliers, chi-square-based probabilities were first derived identifying 48 mothers and 44 fathers as statistical outliers. Their MD values are presented in Figure 1. We then visually inspected the distribution of these values and observed a marked increase at the upper tail. Based on this, we excluded participants with MD values exceeding 73, which was considered to represent a disproportionate deviation. This led to the exclusion of five mothers and ten fathers.

Figure 1: Identification and exclusion of outliers based on Mahalanobis distances


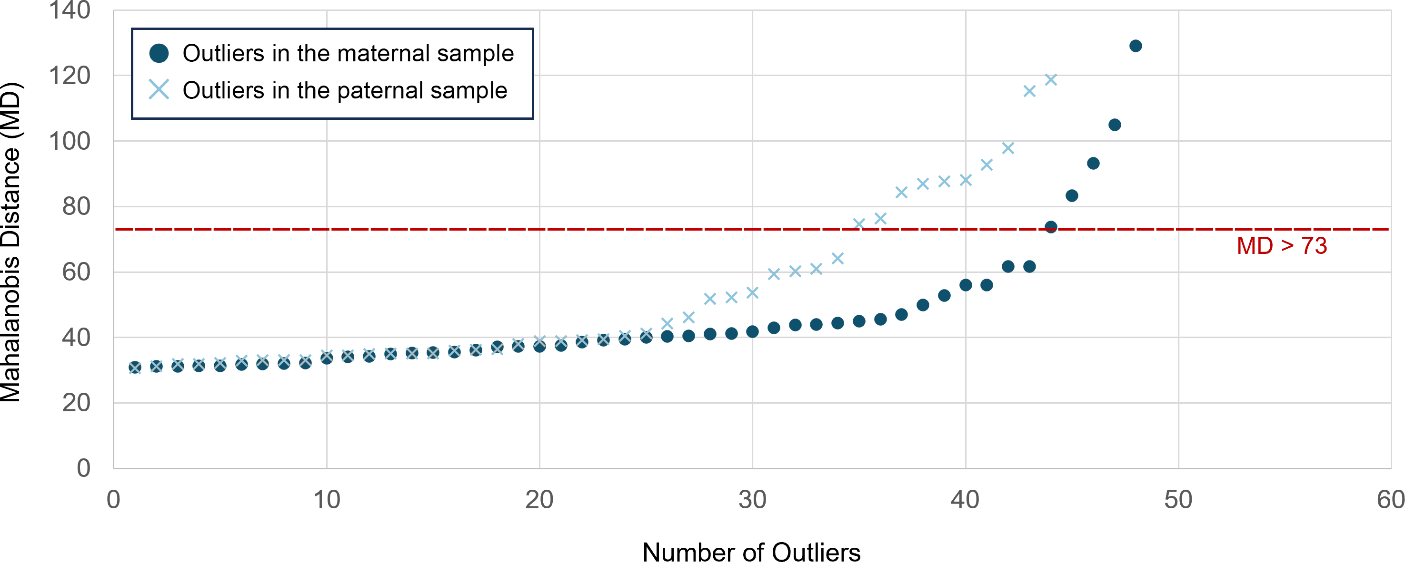

*Note.* The outliers in the figure were arranged in ascending order based on their Mahalanobis distances (MD). Outliers with Mahalanobis distances exceeding 73 were excluded from the analysis.

To assess the robustness of our multinomial logistic regression findings, we re-estimated all models including previously excluded multivariate outliers (see Tables 1 and 2). Overall, the inclusion of these cases did not materially alter the results: odds ratios and effect directions remained highly consistent across models, and the main predictors continued to show strong and significant associations with class membership.

In the maternal sample, inclusion of outliers slightly increased the strength of the association between anger/hostility symptoms and class membership, which became significant for the comparison between the recovering and low-steady classes (p = .005). All other predictors showed comparable estimates to the original model, with only minimal changes in significance levels.

In the paternal sample, the overall pattern of results also remained stable. However, the association between subjective birth experience and class membership was somewhat attenuated and no longer reached significance when comparing the two smallest subgroups (recovering vs. aggravating, p = .071). This likely reflects limited statistical power due to the small size of the recovering class and the increased variability introduced by the inclusion of extreme cases rather than a substantive change in the underlying relationship.

Taken together, the sensitivity analyses indicate that the findings are robust and not unduly influenced by a small number of extreme observations. Minor shifts in p-values appear to be attributable to class-size imbalance and variance inflation rather than to fundamental differences in model structure.

Table 1: Sensitivity analyses: Multinomial logistic regression results for the maternal sample (with outliers)

|  | “Recovering” vs. “low-steady” | | | |  | “Aggravating” vs. “low-steady” | | | |  | “Recovering” vs. “aggravating” | | | |
| --- | --- | --- | --- | --- | --- | --- | --- | --- | --- | --- | --- | --- | --- | --- |
|  | OR | 95% CI | | p |  | OR | 95% CI | | p |  | OR | 95% CI | | p |
|  |  | LL | UL |  |  |  | LL | UL |  |  |  | LL | UL |  |
| Depressive symptoms | 0.984 | 0.906 | 1.070 | .710 |  | 0.924 | 0.844 | 1.010 | .083 |  | 1.066 | 0.952 | 1.194 | .270 |
| Anxiety symptoms | 1.001 | 0.897 | 1.118 | .981 |  | 0.974 | 0.872 | 1.090 | .650 |  | 1.028 | 0.885 | 1.193 | .721 |
| Somatization symptoms | 0.960 | 0.891 | 1.035 | .293 |  | 1.056 | 0.979 | 1.140 | .160 |  | 0.909 | 0.825 | 1.002 | .055 |
| Anger/hostility symptoms | 1.168 | 1.049 | 1.301 | .005* |  | 1.190 | 1.055 | 1.343 | .005* |  | 0.981 | 0.850 | 1.133 | .798 |
| Subjective birth experience | 0.964 | 0.950 | 0.980 | .000** |  | 0.981 | 0.969 | 0.994 | .004* |  | 0.983 | 0.973 | 1.104 | .077 |
| Difficult child temperament | 1.143 | 1.096 | 1.191 | .000** |  | 1.103 | 1.047 | 1.161 | .000** |  | 1.036 | 0.973 | 1.104 | .271 |
| Relationship Satisfaction | 0.998 | 0.929 | 1.072 | .953 |  | 0.952 | 0.875 | 1.035 | .249 |  | 1.048 | 0.946 | 1.162 | .369 |
| First-time parent | 0.584 | 0.197 | 1.731 | .332 |  | 0.427 | 0.126 | 1.442 | .170 |  | 1.369 | 0.280 | 6.705 | .698 |
| Age | 1.016 | 0.940 | 1.098 | .683 |  | 0.949 | 0.869 | 1.036 | .241 |  | 1.071 | 0.959 | 1.196 | .223 |
| Education (>10 years vs. ≤10 years) | 1.474 | 0.581 | 3.740 | .414 |  | 2.393 | 0.587 | 9.752 | .223 |  | 0.616 | 0.124 | 3.060 | .554 |

*Note.* Two sets of results were obtained for each pair of classes (i.e., “aggravating” vs. “low-steady” and “low-steady” vs. “aggravating”), yielding identical findings but with ORs in opposite directions. To prevent redundancy, only one comparison per pair of classes is displayed. LL = lower limit; UL = upper limit.
** p < .01 (2-tailed). ** p < .001 (2-tailed).*

Table 2: Sensitivity analyses: Multinomial logistic regression results for the paternal sample (with outliers)

|  | “Recovering” vs. “low-steady” | | | |  | “Aggravating” vs. “low-steady” | | | |  | “Recovering” vs. “aggravating” | | | |
| --- | --- | --- | --- | --- | --- | --- | --- | --- | --- | --- | --- | --- | --- | --- |
|  | OR | 95% CI | | p |  | OR | 95% CI | | p |  | OR | 95% CI | | p |
|  |  | LL | UL |  |  |  | LL | UL |  |  |  | LL | UL |  |
| Depressive symptoms | 1.323 | 0.802 | 2.182 | .273 |  | 1.222 | 0.767 | 1.946 | .399 |  | 1.083 | 0.602 | 1.950 | .790 |
| Anxiety symptoms | 0.811 | 0.546 | 1.204 | .298 |  | 0.893 | 0.501 | 1.589 | .699 |  | 0.908 | 0.545 | 1.513 | .712 |
| Somatization symptoms | 0.601 | 0.317 | 1.140 | .119 |  | 0.930 | 0.458 | 1.889 | .840 |  | 0.647 | 0.284 | 1.470 | .298 |
| Anger/hostility symptoms | 1.543 | 0.939 | 2.536 | .087 |  | 1.205 | 0.896 | 1.620 | .217 |  | 1.281 | 0.809 | 2.029 | .292 |
| Subjective birth experience | 0.402 | 0.284 | 0.569 | .000** |  | 0.631 | 0.435 | 0.917 | .016* |  | 0.637 | 0.390 | 1.040 | .071 |
| Difficult child temperament | 4.262 | 2.719 | 6.680 | .000** |  | 2.088 | 1.329 | 3.281 | .001* |  | 2.041 | 1.128 | 3.692 | .018* |
| Relationship Satisfaction | 0.896 | 0.601 | 1.337 | .592 |  | 0.857 | 0.569 | 1.292 | .462 |  | 1.046 | 0.620 | 1.765 | .867 |
| First-time parent | 3.773 | 1.351 | 10.532 | .011* |  | 2.392 | 0.811 | 7.057 | .114 |  | 1.577 | 0.425 | 5.846 | .495 |
| Age | 0.906 | 0.831 | 0.988 | .026* |  | 0.913 | 0.840 | 0.993 | .033* |  | 0.992 | 0.890 | 1.106 | .886 |

*Note.* Two sets of results were obtained for each pair of classes (i.e., “aggravating” vs. “low-steady” and “low-steady” vs. “aggravating”), yielding identical findings but with ORs in opposite directions. To prevent redundancy, only one comparison per pair of classes is displayed. LL = lower limit; UL = upper limit.
** p < .01 (2-tailed). ** p < .001 (2-tailed).*
