# Supplementary material for: Understanding parental bonding in the first two years after birth: exploring family predictors using growth mixture modeling
Source: BMC Psychol. 2026 May 27;14:784. doi: 10.1186/s40359-026-04788-9 (PMC13214287; doi:10.1186/s40359-026-04788-9)
Supplement: Supplementary file 3 — Supplementary Material 3. [file 40359_2026_4788_MOESM3_ESM.docx]

# Rationale for exclusion of parents of multiples

This file provides supplementary methodological details. References specific to this file are listed at the end.

While our own descriptive comparisons revealed no notable prepartum differences in mental health indicators between parents of multiples and singletons (data not shown but available upon request), the postpartum context of multiple births differs substantially. Prior research has shown that multiple-birth families constitute a clinically and contextually distinct subgroup characterized by factors that can directly affect bonding processes, such as reduced maternal sensitivity and responsiveness in monochorionic twins (1), lower bonding and sleep quality and higher parenting stress (2), elevated risk of postpartum depression (3,4), more frequent and longer neonatal intensive care stays (5), and reduced individual time and face-to-face interaction with each infant (6). Given these well-documented differences and the small number of multiple-birth parents who provided data on both bonding (at least one postpartum time point) and predictor variables in our sample (*n* = 41), we decided to exclude this subgroup to maintain conceptual and methodological coherence in modeling bonding trajectories.

# References

1. Ionio C, Mascheroni E, Lista G, Colombo C, Ciuffo G, Landoni M, et al. Monochorionic Twins and the Early Mother-Infant Relationship: An Exploratory Observational Study of Mother-Infant Interaction in the Post-Partum Period. Int J Environ Res Public Health. 2022;19(5).

2. Wenze SJ, Battle CL, Huntley ED, Gaugler TL, Kats D. Ecological momentary assessment of postpartum outcomes in mothers of multiples: lower maternal-infant bonding, higher stress, and more disrupted sleep. Arch Womens Ment Health. 2023;26(3):361–78.

3. Choi Y, Bishai D, Minkovitz CS. Multiple births are a risk factor for postpartum maternal depressive symptoms. Pediatrics [Internet]. 2009;123(4):1147–54. Available from: http://www.embase.com/search/results?subaction=viewrecord&from=export&id=L354628911%0Ahttp://pediatrics.aappublications.org/cgi/reprint/123/4/1147%0Ahttp://dx.doi.org/10.1542/peds.2008-1619

4. Egsgaard S, Bliddal M, Lund LC, Vigod SN, Munk-Olsen T. Risk and timing of postpartum depression in parents of twins compared to parents of singletons. Acta Psychiatr Scand. 2025;151(2):163–72.

5. Wright D, Wright A, Rehal A, Syngelaki A, Kristensen SE, Petersen OB, et al. Incidence of neonatal morbidity in small-for-gestational-age twins based on singleton and twin charts. Ultrasound Obstet Gynecol. 2024;63(3):365–70.

6. Holditch-Davis D, Roberts D, Sandelowski M. Early parental interactions with and perceptions of multiple birth infants. J Adv Nurs [Internet]. 1999;30(1):200–10. Available from: http://ovidsp.ovid.com/ovidweb.cgi?T=JS&PAGE=reference&D=emed4&NEWS=N&AN=10403997
