# Supplementary material for: Understanding parental bonding in the first two years after birth: exploring family predictors using growth mixture modeling
Source: BMC Psychol. 2026 May 27;14:784. doi: 10.1186/s40359-026-04788-9 (PMC13214287; doi:10.1186/s40359-026-04788-9)
Supplement: Supplementary file 4 — Supplementary Material 4. [file 40359_2026_4788_MOESM4_ESM.docx]

Table 1: Results of Exclusion Analysis in the Maternal Sample

|  | | Levene's test | | T-Test for equality of means | | | | | | |
| --- | --- | --- | --- | --- | --- | --- | --- | --- | --- | --- |
|  |  | F | *p* | T | df | *p* | Mean difference | SE diff | 95% CI | |
|  |  |  |  |  |  |  |  |  | LL | UL |
| Depressive symptoms | Variances are equal | 2.466 | .117 | -1.203 | 1873 | .229 | -0.457 | 0.380 | -1.202 | 0.288 |
|  | Variances are not equal |  |  | -1.111 | 131.676 | .268 | -0.457 | 0.411 | -1.271 | 0.357 |
| Anxiety symptoms | Variances are equal | 2.605 | .107 | -1.492 | 1871 | .136 | -0.430 | 0.288 | -0.996 | 0.135 |
|  | Variances are not equal |  |  | -1.255 | 126.607 | .212 | -0.430 | 0.343 | -1.109 | 0.248 |
| Obsessive-compulsive symptoms | Variances are equal | 0.377 | .539 | -0.613 | 1876 | .540 | -0.232 | 0.379 | -0.976 | 0.511 |
|  | Variances are not equal |  |  | -0.543 | 134 | .588 | -0.232 | 0.428 | -1.079 | 0.615 |
| Somatization symptoms | Variances are equal | 0.177 | .674 | -0.506 | 1872 | .613 | -0.222 | 0.440 | -1.085 | 0.640 |
|  | Variances are not equal |  |  | -0.455 | 129.642 | .650 | -0.222 | 0.489 | -1.190 | 0.745 |
| Anger/hostility symptoms | Variances are equal | 15.631*** | .000 | -2.733** | 1872 | .006 | -0.568 | 0.208 | -0.975 | -0.160 |
|  | Variances are not equal |  |  | -2.120* | 125.915 | .036 | -0.568 | 0.268 | -1.098 | -0.038 |
| Subjective birth experience | Variances are equal | 0.333 | .564 | -0.038 | 1848 | .970 | -0.083 | 2.195 | -4.388 | 4.222 |
|  | Variances are not equal |  |  | -0.039 | 103.832 | .969 | -0.083 | 2.139 | -4.324 | 4.158 |
| Difficult child temperament | Variances are equal | 1.491 | .222 | -1.520 | 1848 | .129 | -1.294 | 0.852 | -2.965 | 0.376 |
|  | Variances are not equal |  |  | -1.427 | 101.842 | .157 | -1.294 | 0.907 | -3.094 | 0.505 |
| Relationship satisfaction | Variances are equal | 0.358 | .550 | -0.085 | 1853 | .932 | -0.036 | 0.418 | -0.855 | 0.784 |
|  | Variances are not equal |  |  | -0.077 | 106.947 | .939 | -0.036 | 0.464 | -0.955 | 0.884 |
| First-time parent | Variances are equal | 6.400* | .011 | 1.186 | 1867 | .236 | 0.045 | 0.038 | -0.030 | 0.121 |
|  | Variances are not equal |  |  | 1.296 | 130.260 | .197 | 0.045 | 0.035 | -0.024 | 0.115 |
| Age | Variances are equal | 1.411 | .235 | -0.087 | 1867 | .931 | -0.033 | 0.381 | -0.780 | 0.714 |
|  | Variances are not equal |  |  | -0.093 | 129.368 | .926 | -0.033 | 0.356 | -0.738 | 0.672 |
| Education (>10 years vs. <=10 years) | Variances are equal | 1.177 | 0.278 | -0.526 | 1877 | .599 | -0.020 | 0.038 | -0.095 | 0.055 |
|  | Variances are not equal |  |  | -0.541 | 140.872 | .589 | -0.020 | 0.037 | -0.094 | 0.053 |

*Note*. SE diff = Standard Error of the Difference; LL = lower limit; UL = upper limit.

** p < .05 (two-tailed). ** p < .01 (two-tailed). *** p < .001 (two-tailed).*

Table 2: Results of Exclusion Analysis in the Paternal Sample

|  | | Levene's test | | T-Test for equality of means | | | | | | |
| --- | --- | --- | --- | --- | --- | --- | --- | --- | --- | --- |
|  |  | F | p | T | df | p | Mean difference | SE diff | 95% CI | |
|  |  |  |  |  |  |  |  |  | LL | UL |
| Depressive symptoms | Variances are equal | 3.001 | .083 | -2.014* | 1271 | .044 | -0.593 | 0.294 | -1.170 | -0.015 |
|  | Variances are not equal |  |  | -1.840 | 189.138 | .067 | -0.593 | 0.322 | -1.228 | 0.043 |
| Anxiety symptoms | Variances are equal | 0.030 | .862 | 0.804 | 1264 | .421 | 0.154 | 0.192 | -0.222 | 0.531 |
|  | Variances are not equal |  |  | 0.804 | 187.991 | .423 | 0.154 | 0.192 | -0.225 | 0.534 |
| Obsessive-compulsive symptoms | Variances are equal | 0.023 | .880 | 1.202 | 1266 | .229 | 0.368 | 0.306 | -0.233 | 0.969 |
|  | Variances are not equal |  |  | 1.189 | 189.951 | .236 | 0.368 | 0.310 | -0.243 | 0.979 |
| Somatization symptoms | Variances are equal | 0.148 | .701 | -0.234 | 1264 | .815 | -0.054 | 0.231 | -0.506 | 0.399 |
|  | Variances are not equal |  |  | -0.233 | 187.605 | .816 | -0.054 | 0.232 | -0.511 | 0.403 |
| Anger/hostility symptoms | Variances are equal | 0.877 | .349 | -0.598 | 1263 | .550 | -0.102 | 0.171 | -0.438 | 0.234 |
|  | Variances are not equal |  |  | -0.562 | 180.321 | .575 | -0.102 | 0.182 | -0.461 | 0.257 |
| Subjective birth experience | Variances are equal | 2.056 | .152 | -1.411 | 1205 | .158 | -2.325 | 1.648 | -5.558 | 0.907 |
|  | Variances are not equal |  |  | -1.572 | 106.891 | .119 | -2.325 | 1.479 | -5.257 | 0.607 |
| Dfficult child temperament | Variances are equal | 0.057 | .811 | 1.470 | 1257 | .142 | 1.013 | 0.689 | -0.339 | 2.364 |
|  | Variances are not equal |  |  | 1.476 | 177.615 | .142 | 1.013 | 0.686 | -0.341 | 2.366 |
| Relationship satisfaction | Variances are equal | 0.133 | .715 | 0.254 | 1269 | .799 | 0.089 | 0.351 | -0.600 | 0.779 |
|  | Variances are not equal |  |  | 0.254 | 195.971 | .800 | 0.089 | 0.351 | -0.604 | 0.782 |
| First-time parent | Variances are equal | 2.040 | .153 | -0.740 | 1244 | .459 | -0.027 | 0.037 | -0.100 | 0.045 |
|  | Variances are not equal |  |  | -0.710 | 154.315 | .479 | -0.027 | 0.039 | -0.103 | 0.049 |
| Age | Variances are equal | 1.135 | .287 | -2.567** | 1266 | .010 | -1.084 | 0.422 | -1.912 | -0.256 |
|  | Variances are not equal |  |  | -2.455* | 186.332 | .015 | -1.084 | 0.441 | -1.955 | -0.213 |
| Education (>10 years vs. <=10 years) | Variances are equal | 8.052** | .005 | 1.591 | 1268 | .112 | 0.062 | 0.039 | -0.014 | 0.138 |
|  | Variances are not equal |  |  | 1.520 | 189.224 | .130 | 0.062 | 0.041 | -0.018 | 0.142 |

*Note*. SE diff = Standard Error of the Difference; LL = lower limit; UL = upper limit.

** p < .05 (two-tailed). ** p < .01 (two-tailed). *** p < .001 (two-tailed).*
