# Supplementary material for: Understanding parental bonding in the first two years after birth: exploring family predictors using growth mixture modeling
Source: BMC Psychol. 2026 May 27;14:784. doi: 10.1186/s40359-026-04788-9 (PMC13214287; doi:10.1186/s40359-026-04788-9)
Supplement: Supplementary file 5 — Supplementary Material 5. [file 40359_2026_4788_MOESM5_ESM.docx]

Table 1: Class counts, estimated proportions, and average posterior probabilities for the final class solutions in the maternal sample

|  | **Class 1** | **Class 2** | **Class 3** | **Class 4** | **Class 5** |
| --- | --- | --- | --- | --- | --- |
| **1 class solution** | | | | | |
| Class count | 1,756 | - | - | - | - |
| Class proportions | 1.000 | - | - | - | - |
| AvePP | 1.000 | - | - | - | - |
| **2 class solution** |  |  |  |  |  |
| Class count | 1,681 | 75 | - | - | - |
| Class proportions | 0.957 | 0.042 | - | - | - |
| AvePP | 0.994 | 0.925 | - | - | - |
| **3 class solution** |  |  |  |  |  |
| Class count | 1,640 | 61 | 55 | - | - |
| Class proportions | 0.933 | 0.034 | 0.031 | - | - |
| AvePP | 0.991 | 0.901 | 0.926 | - | - |
| **4 class solution** |  |  |  |  |  |
| Class count | 1,523 | 162 | 46 | 25 | - |
| Class proportions | 0.867 | 0.092 | 0.026 | 0.014 | - |
| AvePP | 0.970 | 0.821 | 0.891 | 0.965 | - |
| **5 class solution** |  |  |  |  |  |
| Class count | 1,493 | 184 | 46 | 25 | 8 |
| Class proportions | 0.850 | 0.104 | 0.026 | 0.014 | 0.004 |
| AvePP | 0.968 | 0.808 | 0.908 | 0.959 | 0.974 |

*Note.* AvePP, average posterior probabilities. Classes are ordered by descending class size (largest to smallest).

Table 2: Class counts, estimated proportions, and average posterior probabilities for the final class solutions in the paternal sample

|  | **Class 1** | **Class 2** | **Class 3** | **Class 4** | **Class 5** |
| --- | --- | --- | --- | --- | --- |
| **1 class solution** | | | | | |
| Class count | 1,118 | - | - | - | - |
| Class proportions | 1.000 | - | - | - | - |
| AvePP | 1.000 | - | - | - | - |
| **2 class solution** |  |  |  |  |  |
| Class count | 1,077 | 41 | - | - | - |
| Class proportions | 0.963 | 0.036 | - | - | - |
| AvePP | 0.991 | 0.879 | - | - | - |
| **3 class solution** |  |  |  |  |  |
| Class count | 1,030 | 56 | 32 | - | - |
| Class proportions | 0.921 | 0.050 | 0.028 | - | - |
| AvePP | 0.980 | 0.858 | 0.900 | - | - |
| **4 class solution** |  |  |  |  |  |
| Class count | 999 | 60 | 45 | 14 | - |
| Class proportions | 0.893 | 0.053 | 0.040 | 0.012 | - |
| AvePP | 0.970 | 0.795 | 0.865 | 0.920 | - |
| **5 class solution** |  |  |  |  |  |
| Class count | 973 | 92 | 34 | 13 | 6 |
| Class proportions | 0.870 | 0.082 | 0.030 | 0.011 | 0.005 |
| AvePP | 0.955 | 0.771 | 0.899 | 0.928 | 0.913 |

*Note.* AvePP, average posterior probabilities. Classes are ordered by descending class size (largest to smallest).

Figure 1: Estimated trajectory means of the 1-class solution within the maternal sample


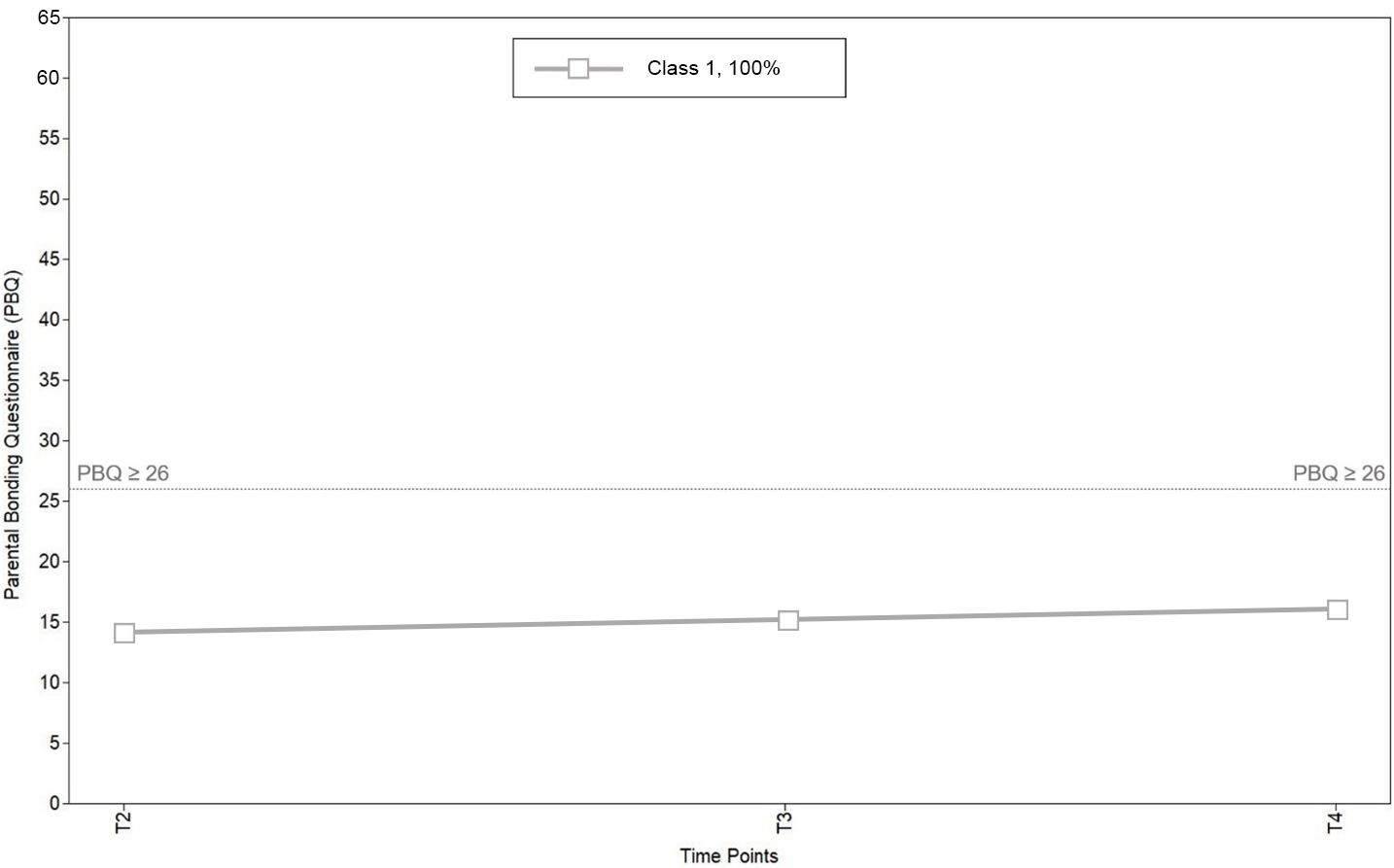

*Note.* T2 = around eight weeks after the anticipated birth date; T3 = around 14 months after the actual birth date; T4 = around two years after the actual birth date.

Figure 2: Estimated trajectory means of the 2-class solution within the maternal sample


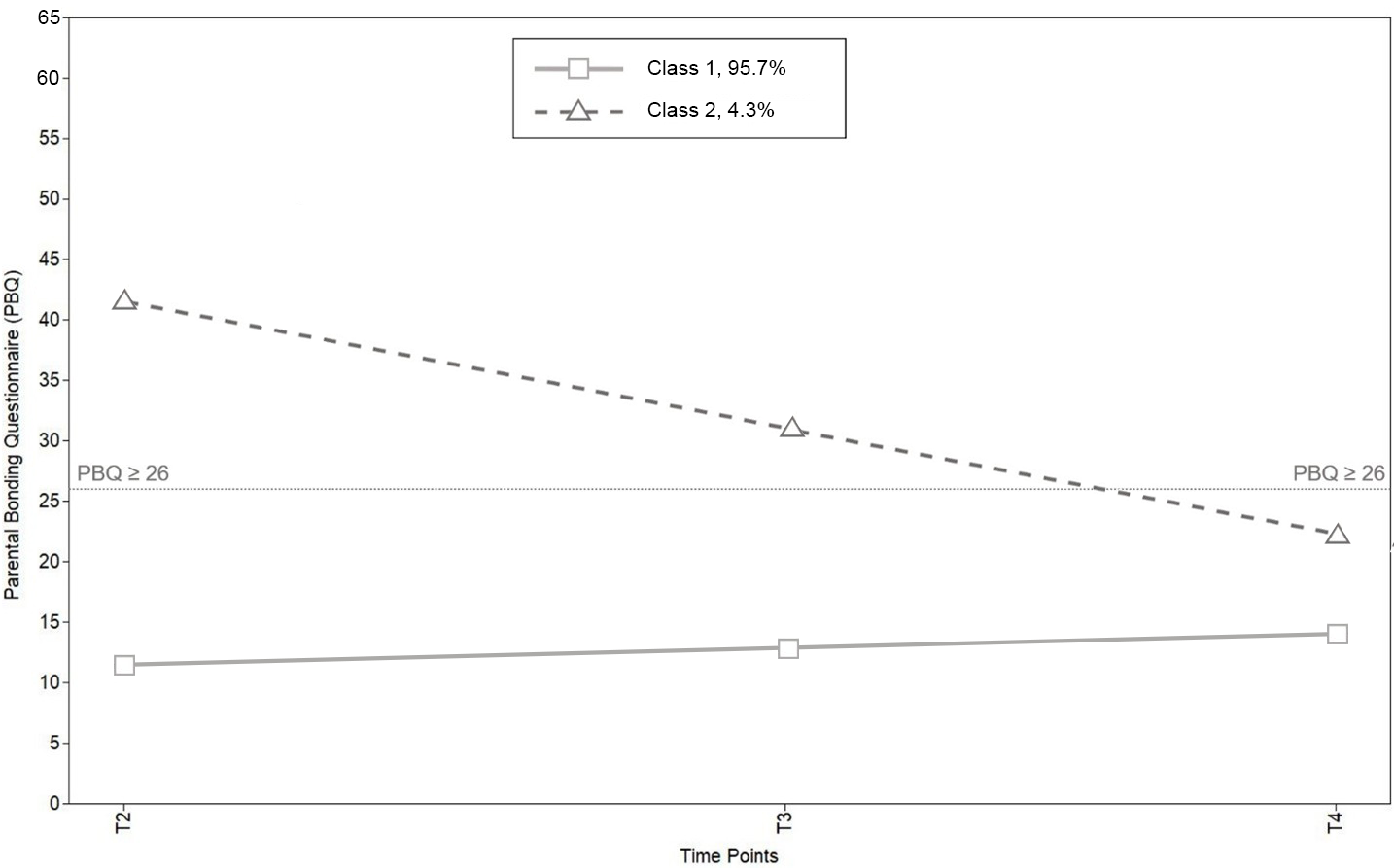


*Note.* T2 = around eight weeks after the anticipated birth date; T3 = around 14 months after the actual birth date; T4 = around two years after the actual birth date.

Figure 3: Estimated trajectory means of the 3-class solution within the maternal sample


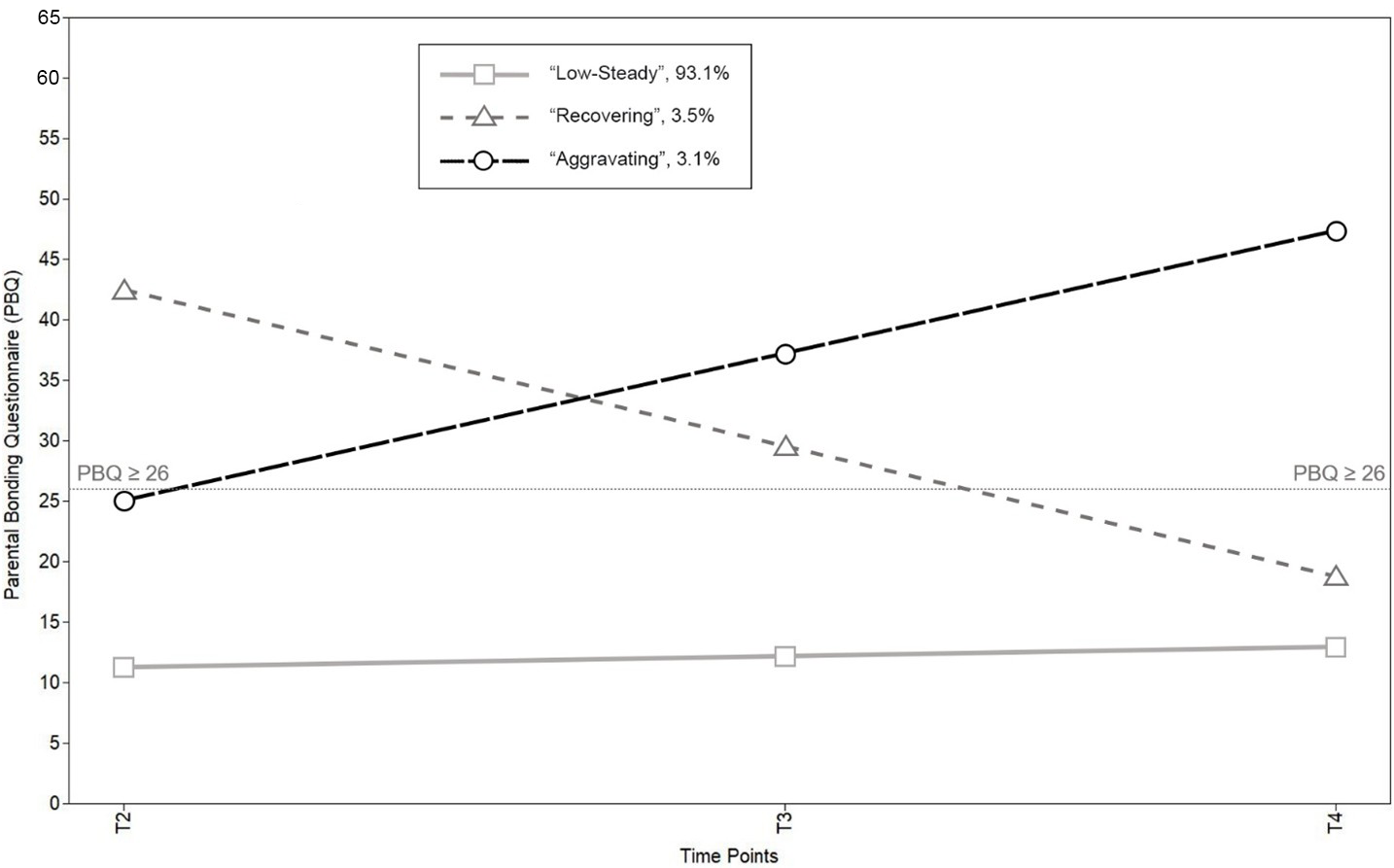


*Note.* T2 = around eight weeks after the anticipated birth date; T3 = around 14 months after the actual birth date; T4 = around two years after the actual birth date.

Figure 4: Estimated trajectory means of the 4-class solution within the maternal sample


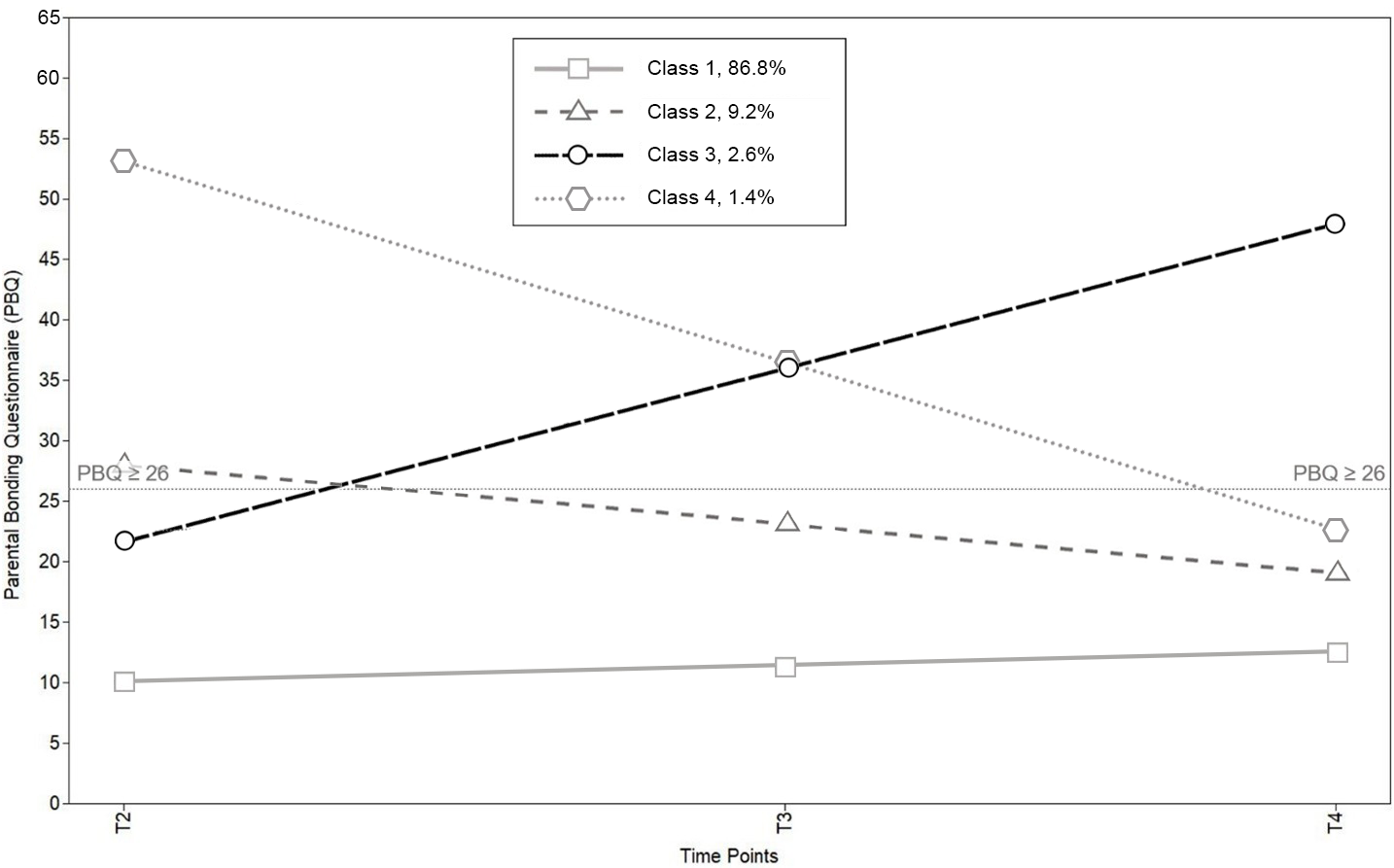


*Note.* T2 = around eight weeks after the anticipated birth date; T3 = around 14 months after the actual birth date; T4 = around two years after the actual birth date.

Figure 5: Estimated trajectory means of the 5-class solution within the maternal sample


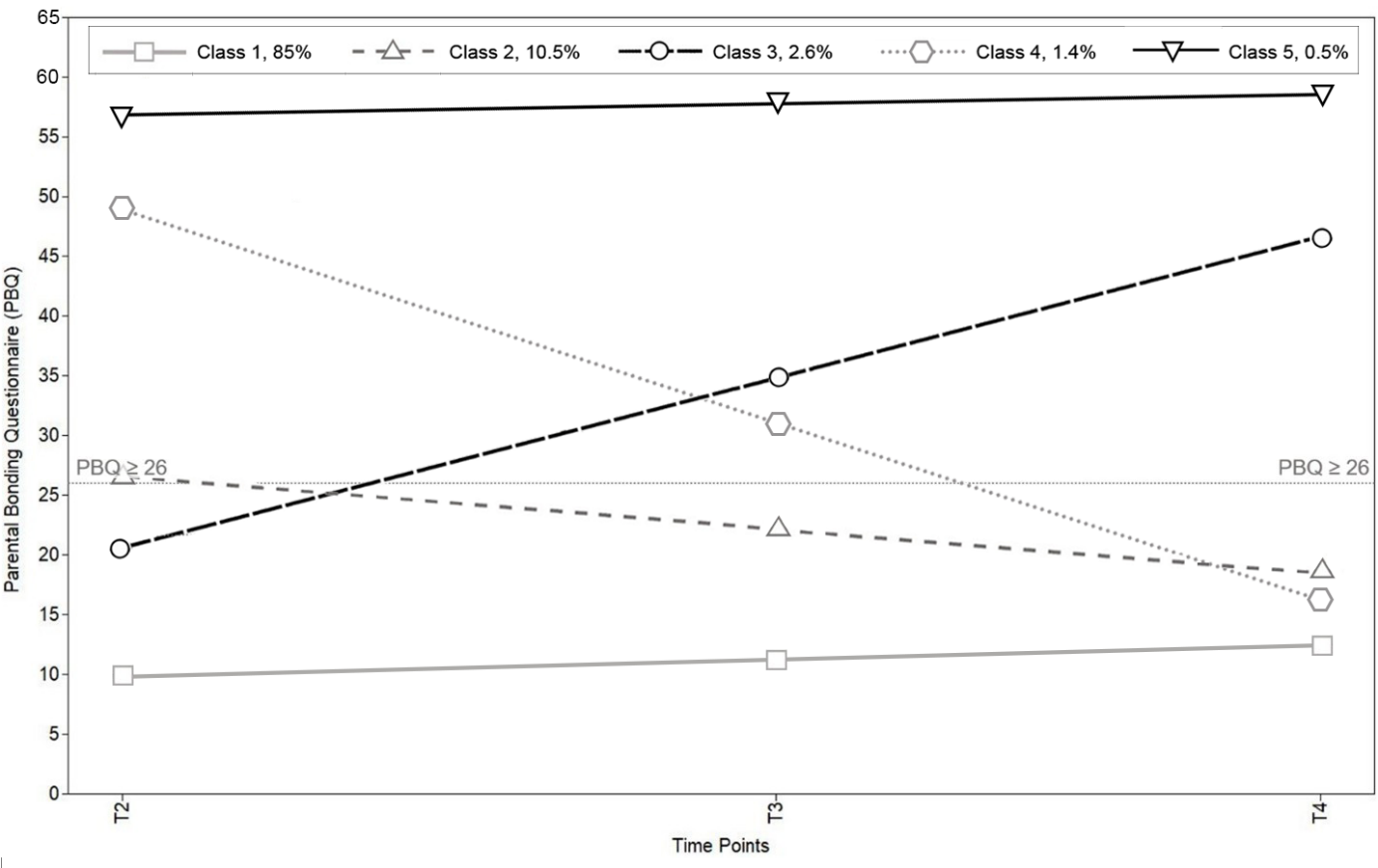


*Note.* T2 = around eight weeks after the anticipated birth date; T3 = around 14 months after the actual birth date; T4 = around two years after the actual birth date.

Figure 6: Estimated trajectory means of the 1-class solution within the paternal sample


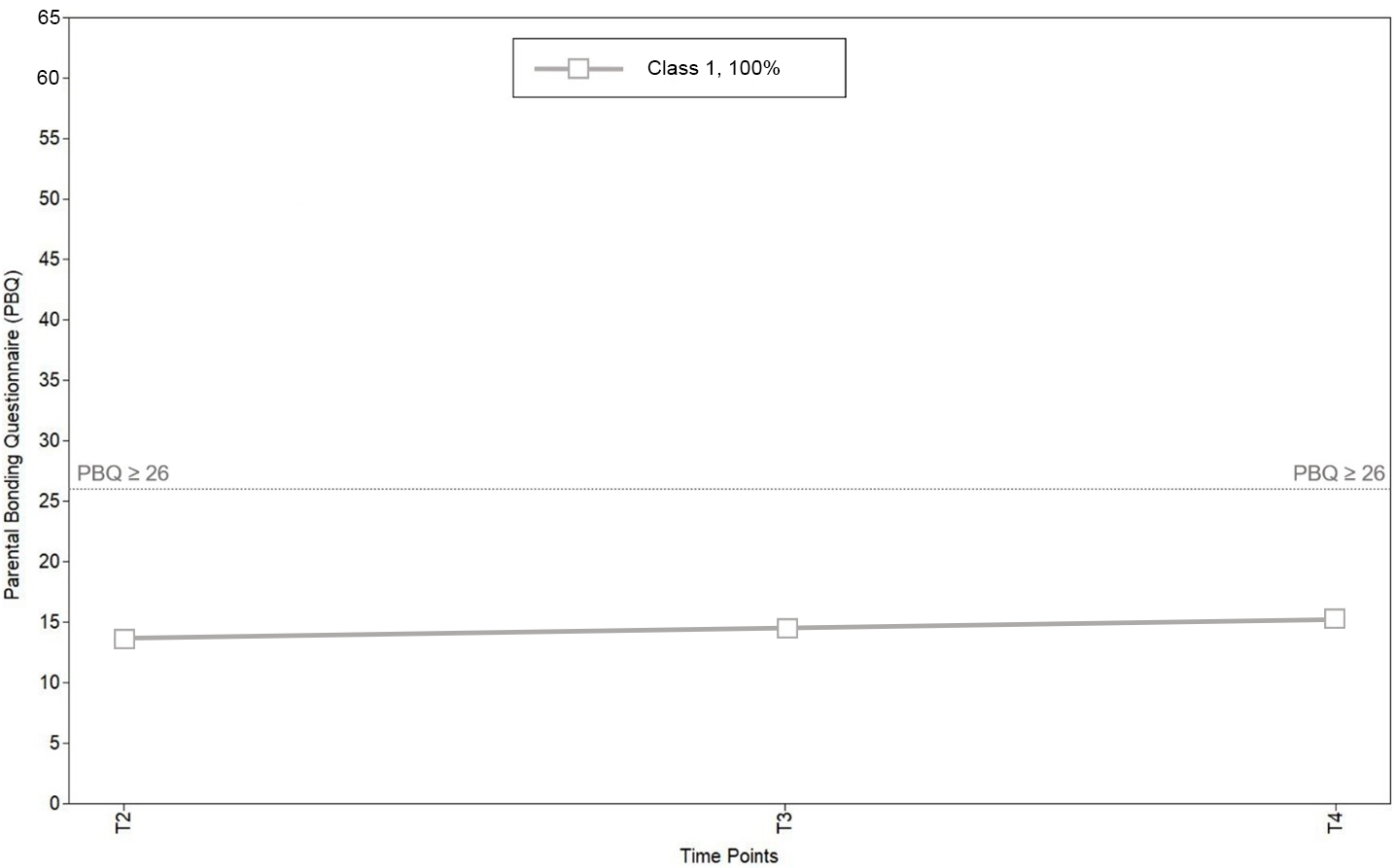


*Note.* T2 = around eight weeks after the anticipated birth date; T3 = around 14 months after the actual birth date; T4 = around two years after the actual birth date.

Figure 7: Estimated trajectory means of the 2-class solution within the paternal sample


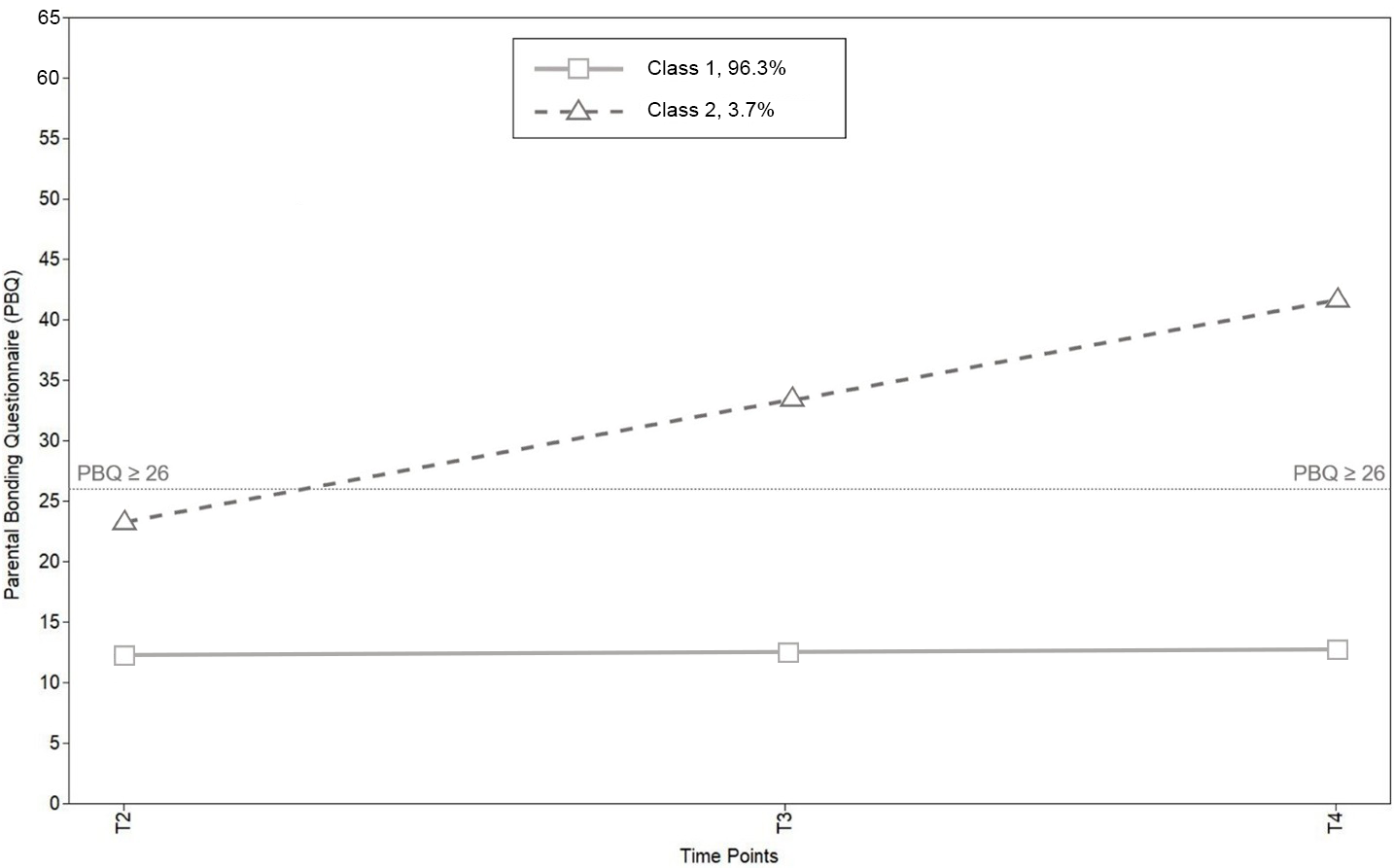
 *Note.* T2 = around eight weeks after the anticipated birth date; T3 = around 14 months after the actual birth date; T4 = around two years after the actual birth date.

Figure 8: Estimated trajectory means of the 3-class solution within the paternal sample


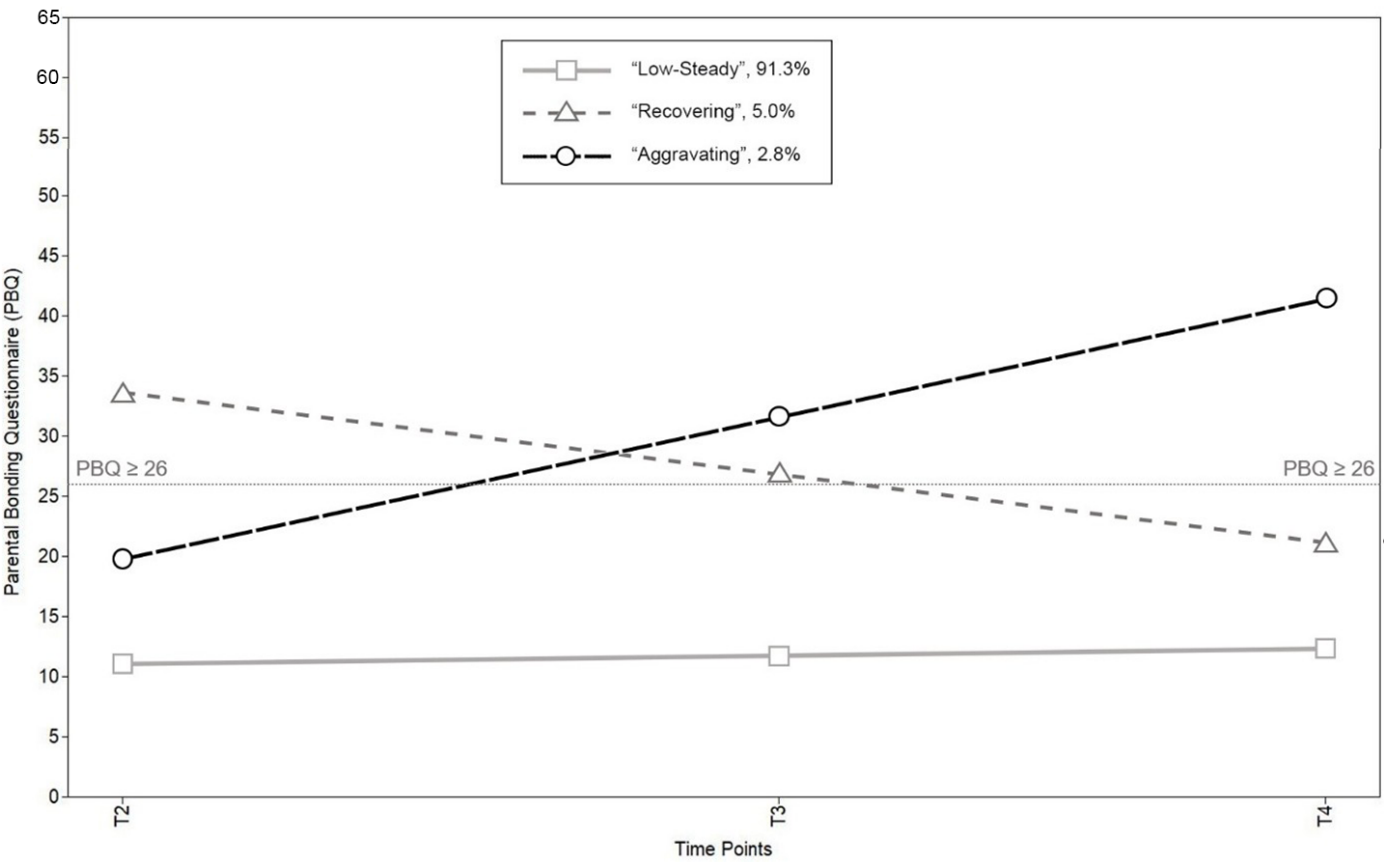


*Note.* T2 = around eight weeks after the anticipated birth date; T3 = around 14 months after the actual birth date; T4 = around two years after the actual birth date.

Figure 9: Estimated trajectory means of the 4-class solution within the paternal sample


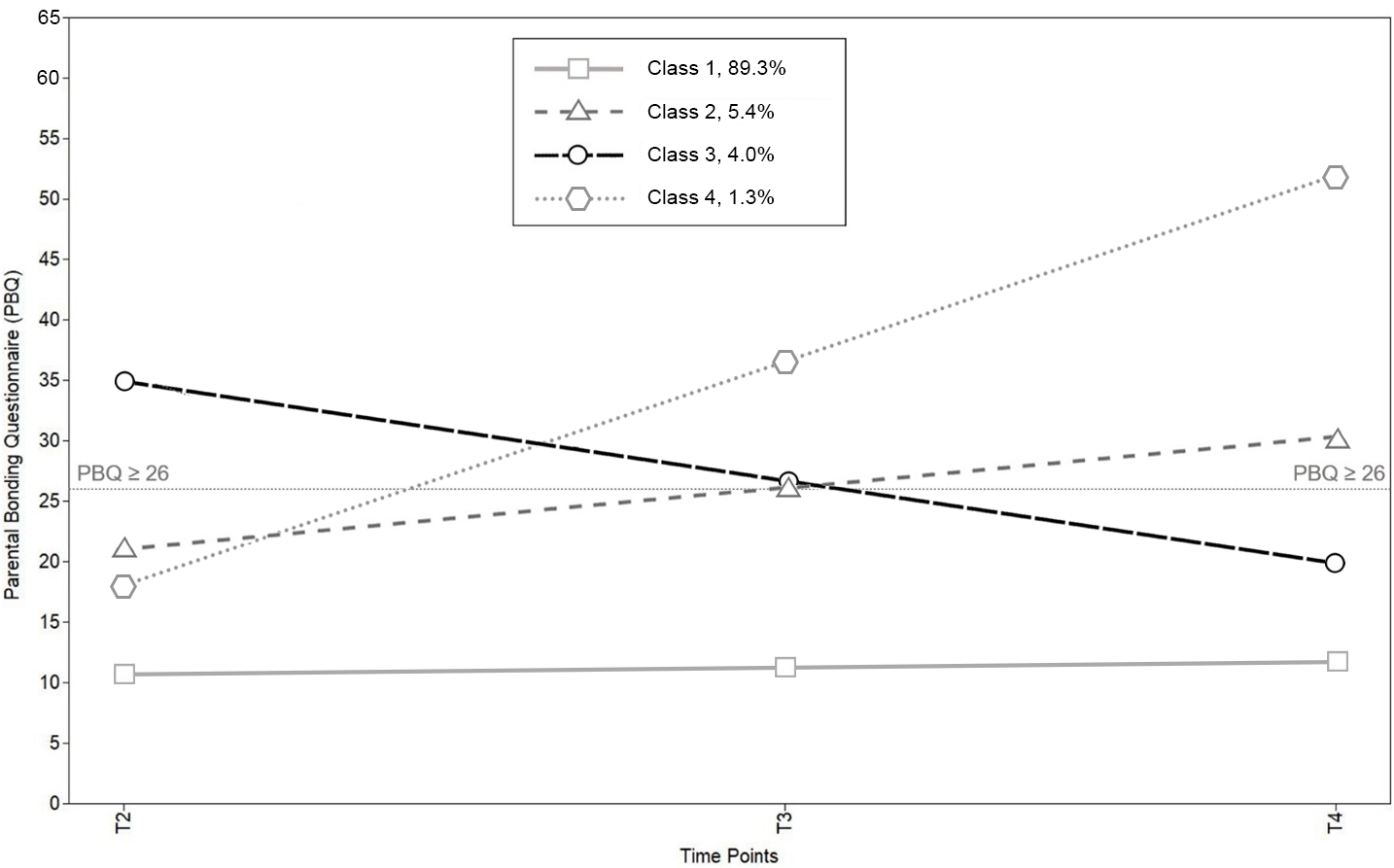


*Note.* T2 = around eight weeks after the anticipated birth date; T3 = around 14 months after the actual birth date; T4 = around two years after the actual birth date.

Figure 10: Estimated trajectory means of the 5-class solution within the paternal sample


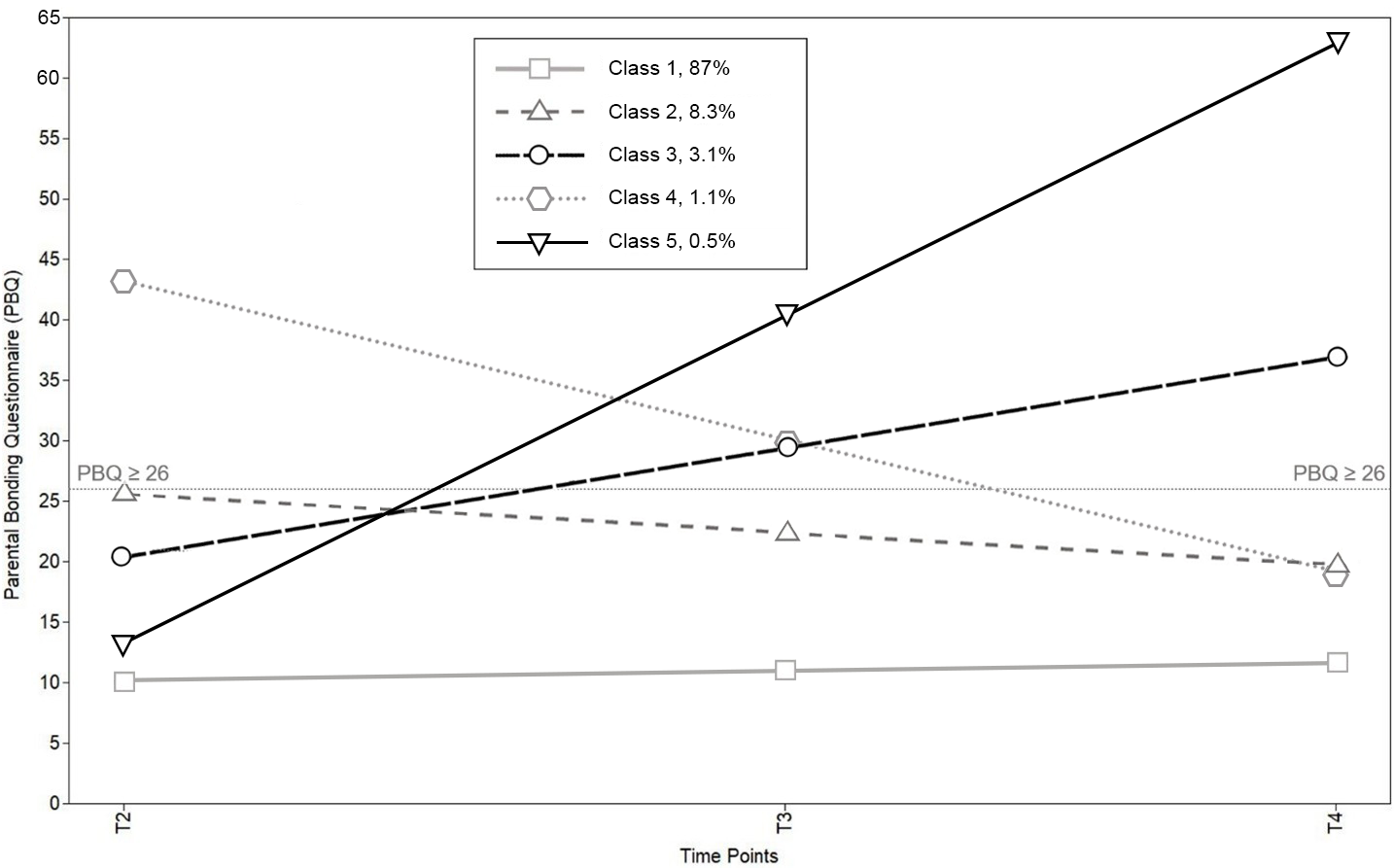
 *Note.* T2 = around eight weeks after the anticipated birth date; T3 = around 14 months after the actual birth date; T4 = around two years after the actual birth date.
